# Orolabial and Genital Herpes Clinical Trials: A Meta-analysis of Endpoints

**DOI:** 10.1093/ofid/ofaf776

**Published:** 2025-12-17

**Authors:** Abigail Sloan, Mahta Mortezavi, Jacqueline Gerhart, Anindita Banerjee, Negar Niki Alami, Isabel Najera, Sima Ahadieh, Alexis Bernard Dalam, Joshua T Schiffer, Rajul Patel, Christine Johnston

**Affiliations:** Research and Development, Pfizer, New York City, New York, USA; Research and Development, Kymera Therapeutics, Watertown, Massachusetts, USA; Research and Development, Pfizer, New York City, New York, USA; Research and Development, Pfizer, New York City, New York, USA; Research and Development, Pfizer, New York City, New York, USA; Research and Development, Faes Farma, Leioa, Spain; Research and Development, Pfizer, New York City, New York, USA; Research and Development, Pfizer, New York City, New York, USA; Department of Medicine, University of Washington, Seattle, Washington, USA; Vaccine and Infectious Disease Division, Fred Hutchinson Cancer Center, Seattle, Washington, USA; Department of Medicine, University of Southampton, Southampton, UK; Vaccine and Infectious Disease Division, Fred Hutchinson Cancer Center, Seattle, Washington, USA; Departments of Medicine and Laboratory Medicine, University of Washington, Seattle, Washington, USA

**Keywords:** genital herpes, HSV, meta-analysis, orolabial herpes, systematic review

## Abstract

Although several antiviral agents are licensed for the treatment of orolabial and genital herpes simplex virus infections, new therapies are needed. Trial design is challenging for these indications due to the heterogeneity of endpoints in prior trials. We conducted a systematic review and meta-analysis of randomized placebo-controlled trials published between 1995 and 2024 consisting of adults with established herpes simplex virus infection who were immunocompetent and nonpregnant. A total of 22 articles met the inclusion criteria. For episodic treatment, endpoints included time to healing, proportion with an aborted lesion, and time to cessation of symptoms. For daily suppressive therapy, endpoints included time to first recurrence, proportion recurrence-free at 1 year, and total shedding rate. We observed that over the last 30 years, clinical trials have used various endpoints with nonstandardized definitions. A reassessment of appropriate endpoints along with regulatory guidance would assist with consistent study design for evaluation of new agents.

## Teaser

This meta-analysis illustrates the heterogeneity of efficacy endpoints utilized in placebo-controlled trials to test antiviral agents for the treatment and suppression of orolabial and genital herpes and highlights the need for further innovation, standardization, and consistency in trial design.

Herpes simplex virus (HSV) infections, caused by HSV-1 and HSV-2, are widely prevalent worldwide and manifest clinically as recurrent orolabial herpes or recurrent genital herpes [1]. Orolabial herpes is usually caused by HSV-1, although a minority of cases are caused by HSV-2. Recurrent genital herpes is most commonly caused by HSV-2, but HSV-1 has become the leading cause of first-episode genital herpes in some populations [2].

There is a broad spectrum of clinical presentations for orolabial and genital herpes, with some patients remaining asymptomatic and others experiencing significant, frequent, and debilitating episodes [3]. Episodes can be associated with mucocutaneous symptoms and rarely with systemic symptoms (eg, fever, malaise, headaches). During initial infection, the virus establishes latency in the sensory ganglia and can reactivate, traveling along sensory neurons to the skin and other mucosal sites to cause recurrences. Following the first year of infection, recurrences due to either viral type generally decrease in number and severity, although the clinical course varies substantially from patient to patient.

The median genital recurrence rate of HSV-2 is about 4 per year: approximately 40% of patients have at least 6 recurrences in the first year and 20% have >10 recurrences [4]. By contrast, recurrences of genital HSV-1 occur approximately once per year. The mean duration of lesions and duration of viral shedding appear shorter than the first episode (10 vs 19 days of lesions and 2 vs 9 days for viral shedding) [5, 6].

Episodic therapy involves self-administration of antivirals for an acute episode at the onset of prodromal symptoms (tingling, paresthesia, pruritus); it also shortens the time to crusting and healing of lesions and decreases the duration of viral shedding and overall severity as compared with placebo or untreated disease.

Chronic daily suppressive antiviral therapy may be considered for those with frequent/severe recurrences or those who wish to reduce the risk of transmission to a sexual partner uninfected with HSV. Currently available suppressive therapy reduces the risk of HSV reactivation and decreases but does not eliminate shedding in patients with frequent recurrences [7]. Recurrences, viral shedding, and transmission to sexual partners can still occur [6].

The clinical trials leading to the approval of currently available therapies were published as early as the 1980s, with heterogeneous endpoints to measure efficacy in abbreviating symptoms or suppressing recurrences. While regulators have issued some guideline documents, such as the 2017 Food and Drug Administration (FDA) guidance on developing drugs for treatment and prevention of recurrent herpes labialis) [8], no guidance on standardized endpoints has been issued. Having a better understanding of endpoints that have been previously used will help with clinical trial design for new antiviral interventions. Here, we summarize the results of a systematic review and meta-analysis to aggregate the clinical trial endpoints used in the last 30 years to measure the efficacy of approved antivirals for these indications.

## METHODS

### Search Strategy and Study Selection

A systematic review was conducted in major online databases, including BIOSIS Previews, Embase, and Ovid Medline, to identify English-language peer-reviewed articles published between January 1995 and March 2024 that reported randomized, double-blind, placebo-controlled trials in orolabial and genital herpes. The time window was chosen to provide a contemporary view of efficacy data following FDA approval of oral antivirals acyclovir (ACV), famciclovir (FMV), and valacyclovir (VCV).

The target population was otherwise healthy adults without HIV-1, HIV-2, or hepatitis C coinfection who were not immunocompromised, pregnant, or newly diagnosed with herpes within 1 year of the trial. The target intervention was oral antiviral agents for herpes simplex, including but not limited to ACV, FMV, VCV, amenamevir, and pritelivir. A detailed description of the search strategy is presented in Supplementary Table 1. Three authors (A. S., M. M., J. G.) verified eligibility, and C. J. confirmed excluded articles.

### Data Extraction and Endpoints

Data were extracted by 2 independent authors (A. S. and A. B. D.). The following demographics and study characteristics were extracted from each trial as available: age, sex, and race of participants and location and dates of trial, as well as trial duration, treatment arms, number of participants, and inclusion criteria for number of episodes in the previous year. Baseline disease characteristics were also tracked, including time since first herpes episode and reported number of herpes episodes in the previous year (Tables 1 and 2) [9–30]. Supplementary Table 2 describes the data availability for each endpoint by article.

**Table 1.** Study Characteristics of Included Trials

Study	Active Intervention Arms and Dose	No.	Rand Ratio	Required Recurrences per Year^a^	Study Duration, mo	Trial Location	Trial Dates
Episodic orolabial							
Spruance: study 1 (2003) [9]	VCV 2 g bid for 1 d then 1 g bid for 1 d VCV 2 g bid for 1 d Placebo	902	1:1:1	≥3		US	
Spruance: study 2 (2003) [9]	VCV 2 g bid for 1 d then 1 g bid for 1 d	954	1:1:1	≥3		US, CA	
	VCV 2 g bid for 1 d						
	Placebo						
Spruance (2006) [11]	FMV 1.5 g once	701	1:1:1	≥3		US, CA, AUS	Oct 2003 to Jan 2005
	FMV 750 mg bid for 1 d						
	Placebo						
Kawashima (2023) [10]	AMV 1.2 g once	855	1:1			JPN	Sep 2019 to Apr 2021
	Placebo						
Episodic genital							
Tyring (1998) [18]	VCV 1 g bid for 5 d	1200	3:3:1	≥4		EU, US, CA	
	ACV 200 mg 5 times daily for 5 d						
	Placebo						
Wald (2002) [12]	ACV 800 mg tid for 2 d	131	1:1	≥3		US	Aug 2001 to Feb 2002
	Placebo						
Aoki (2006) [13]	FMV 1 g bid for 1 d	329	1:1	≥4		US, CA, DE	
	Placebo						
Sacks (2005) [14]	FMV 500 mg bid for 5 d	308	1:1:1:1	≥4		CA	Oct 1991 to Dec 1992
	FMV 250 mg bid for 5 d						
	FMV 125 mg bid for 5 d						
	Placebo						
Paz-Bailey^b^ (2009) [19]	ACV 400 mg tid for 5 d	150	1:1			SA	Mar 2005 to Dec 2006
	Placebo						
Leone (2010) [15]	FMV 1 g bid for 1 d	299	2:1	≥4		US, SA	Jun 2007 to Mar 2009
	Placebo						
Baeten (2012) [16]	ACV 400 mg tid for 5 d	88	2:1			SA, ZM	Jan 2009 to Dec 2009
	Placebo						
Tyring (2012) [17]	VCV 500 mg bid for 3 d	437	1:1:1:1:1:1	≥4		US	Jun 2007 to Aug 2008
	AMV 1.2 g once						
	AMV 400 mg qd for 3 d						
	AMV 200 mg qd for 3 d						
	AMV 100 mg qd for 3 d						
	Placebo						
Suppressive orolabial							
Baker (2003) [20]	VCV 500 mg qd	98	1:1	≥4	4		1999 to 2000
	Placebo						
Suppressive genital							
Mertz (1997) [25]	FMV 500 mg qd	375	1:1:1:1:1:1	≥6	4	US	
	FMV 250 mg bid						
	FMV 250 mg qd						
	FMV 125 mg bid						
	FMV 125 mg qd						
	Placebo						
Patel (1997) [24]	VCV 500 mg qd	382	3:1	≥8	4	EU, AUS	
	Placebo						
Diaz-Mitoma (1998) [21]	FMV 250 mg tid	455	1:1:1:1	≥6	12	CA, EU	
	FMV 250 mg bid						
	FMV 125 mg tid						
	Placebo						
Reitano (1998) [26]	ACV 400 mg bid	1479	2:2:2:2:2:1	≥6	12	EU, US, AUS	
	VCV 250 mg bid						
	VCV 1 g qd						
	VCV 500 mg qd						
	VCV 250 mg qd						
	Placebo						
Tyring (2003) [22]	FMV 250 mg bid	469	1:1	≥6	12	EU	
	Placebo						
Corey (2004) [23]	VCV 500 mg qd	1484	1:1	<10	8	US, CA, AU, LATAM, AUS	Feb 1998 to Jul 2001
	Placebo						
Sacks (2004) [30]	FMV 250 mg tid	177	1:1:1	≥6	4	CA	
	FMV 125 mg tid						
	Placebo						
Fife (2006) [28]	VCV 1 g qd	152	3:1	≥6	2	US	Jun 2004 to Dec 2004
	Placebo						
Strachan (2011) [29]	ACV 400 mg bid	19	1:1		5	US	
	Placebo						
Wald (2014) [27]	PTV 400 mg weekly	156	1:1:1:1:1	1–9	1	US	Apr 2010 to Dec 2010
	PTV 75 mg qd						
	PTV 25 mg qd						
	PTV 5 mg qd						
	Placebo						
	Placebo						

Blank cells indicate not applicable/not reported.

Abbreviations: ACV, acyclovir; AMV, amenamevir; AU, Austria; AUS, Australia; bid, twice daily; CA, Canada; DE, Germany; EU, European Union; FMV, famciclovir; JPN, Japan; LATAM, Latin America; PTV, pritelivir; qd, daily; SA, South Africa; tid, 3 times daily; VCV, valacyclovir; ZM, Zambia.

^a^Inclusion requirement.

^b^Paz-Bailey reported data from a subset of participants in scope of the meta-analysis; data reported are specific to this subset, where available.

**Table 2.** Demographics and Baseline Disease Characteristics of Included Trials

				Frequency of Recurrences		
Study	Female, %	Median Age, y	White, %	Required Per Year ^a^	At Baseline, Median Episodes/y	Median Time Since First Herpes Episode, y
Episodic orolabial						
Spruance: study 1 (2003) [9]	75	37.5	96	≥3	5	20
Spruance: study 2 (2003) [9]	72	37	91	≥3	6	20
Spruance (2006) [11]	67	39	92	≥3	5	20
Kawashima (2023) [10]	66.9	43			3	
Episodic genital						
Tyring (1998) [18]	52	36		≥4		6.8^b^
Wald (2002) [12]	62	33.5	93	≥3	8	7
Aoki (2006) [13]	71	41	86	≥4	6	
Sacks (2005) [14]	47	32.8		≥4		
Paz-Bailey^c^ (2009) [19]	0					
Leone (2010) [15]	66	37	0	≥4	5	
Baeten (2012) [16]	100	38	0			
Tyring (2012) [17]	70	40^b^	71	≥4	7	10.3^b^
Suppressive orolabial						
Baker (2003) [20]	83	38.75^b^	94	≥4		
Suppressive genital						
Mertz (1997) [25]	100	34^b^	87	≥6	7	
Patel (1997) [24]	29.32	33		≥8		5.1
Diaz-Mitoma (1998) [21]	50.99	36.75^b^		≥6		7^b^
Reitano (1998) [26]	52	34		≥6		5.2
Tyring (2003) [22]	57	37^b^	89	≥6		7.5^b^
Corey (2004) [23]	67	35	89	<10		8
Sacks (2004) [30]	100	32.5^b^	96	≥6		
Fife (2006) [28]	66	40^b^	72	≥6		
Strachan (2011) [29]	100	29				3
Wald (2014) [27]	67	41	74	1–9	4	11

Blank cells indicate not applicable/not reported.

^a^Inclusion requirement.

^b^Reported value is a mean since median was not reported.

^c^Paz-Bailey reported data from a subset of participants in scope of the meta-analysis; data provided in table are specific to this subset, where available.

#### Identification of Endpoints for Episodic Treatment of Orolabial and Genital Herpes

For episodic treatment of orolabial herpes, 3 frequently used endpoints were assessed by meta-analysis: time to healing of lesion, proportion with an aborted lesion, and time to resolution of pain. Time to healing of lesion was assessed among nonaborted (vesicular) lesions and was defined as time from start of treatment to loss of crust [9], re-epithelialization [10], or complete loss of crust and re-epithelialization [11]. Determination of crusting and/or re-epithelialization was based on the clinician's observations and participant diary [9, 11] or was unspecified [10]. In all studies, the proportion of participants with an aborted lesion included those with lesion development that did not progress beyond the papular stage following initiation of treatment. Time to resolution of pain was defined as the time from the start of treatment to cessation of pain as reported by the participant [10] or determined by the clinician's observations and participant's diary [9, 11].

Trials investigating episodic treatment of genital herpes used nominally similar endpoints to those in the orolabial herpes trials, but even more heterogeneity existed in endpoint definitions. Time to healing was defined variously as days with papules, blister, ulcers, or crusts [12]; time to re-epithelialization of nonaborted lesions [13]; time from start of treatment to participant reporting no papules, vesicles, ulcers, or crusts [14]; investigator-assessed time to healing of all nonaborted lesions [15]; re-epithelization of skin [16]; time from start of treatment to re-epithelization of nonaborted lesions [17, 18]; and time to healing assessed by study clinician and participant self-reports [19]. To maximize data for analysis, all studies were included in the meta-analysis. Most studies defined aborted lesions as those not progressing beyond the papular stage. Two studies defined aborted lesions as those not progressing beyond the macular or papular stage [17, 18].

The third endpoint assessed by meta-analysis for episodic treatment of genital herpes was time to resolution of symptoms. To maximize data, all studies reporting a composite symptom outcome were included, regardless of the specific list of individual symptoms tracked. Among studies explicitly listing the symptoms assessed, all cited pain, itching, tingling, and burning [13, 14, 17]. Some studies also noted edema [14] or tenderness [13], while others did not list the specific symptoms assessed [12, 18]. All studies reported a composite time to resolution of all symptoms, while some provided results for individual symptoms [13, 14].

#### Identification of Endpoints for Suppressive Therapy for Orolabial and Genital Herpes

Only 1 article studied suppressive therapy for orolabial herpes, using proportion recurrence-free over 4 months and time to first recurrence from the start of treatment as endpoints [20]. Both endpoints required clinical assessment of the lesional episode to be confirmed as a recurrence.

For suppressive therapy for genital herpes, clinical endpoints assessed by meta-analysis included time to first recurrence and proportion of participants recurrence-free after 1 year. Time to first recurrence was defined as the time from start of treatment until a confirmed episode, where the level of evidence required for confirmation varied across studies. Some studies required symptomatic lesion episodes that were confirmed by clinician/investigator assessment and viral culture [21–23]. Other studies required only clinician confirmation [24], allowed symptomatic episodes with lesions and/or a confirmed positive viral culture [25], or did not specify [26]. Several studies stated that prodromal symptoms were not considered an episode [24, 25] while others were not explicit. Proportion recurrence-free was defined as the proportion of participants who remained recurrence-free, based on the same definitions mentioned previously, from the start of treatment through 1 year.

Four studies reported the virologic endpoint of total shedding rate, defined as the proportion of daily swabs positive for HSV-2, including clinical or subclinical shedding, assessed over 28 days [27], 60 days [23, 28], or 140 days [29].

### Statistical Analyses

Placebo-controlled meta-analysis results for time-to-event and binary endpoints are presented as hazard ratios (HRs) and odds ratios (ORs), respectively. All 95% CIs were taken from the original articles except for instances where 90% CIs were recomputed as 95% CIs, as indicated. Some HRs were inverted to standardize comparison across studies. ORs were estimated from counts provided in the article if not reported. When only the proportion was given, the denominator was assumed to be the same as the primary analysis population, and the numerator was estimated as the nearest whole number after multiplying this denominator by the proportion.

Estimates of the global HR or OR were generated via common and random effects meta-analyses implemented in R (version 4.3.1 with meta package version 6.5-0). For each meta-analysis, *I*^2^ was reported to quantify the degree of between-study heterogeneity, where *I*^2^ ≤ 30%, 30%–50%, 50%–75%, and ≥75% were indicative of low, medium, high, and very high heterogeneity, respectively [31]. A χ^2^ test for estimate homogeneity among trials was performed for each endpoint to provide a *P* value for *I*^2^. An alpha level of .05 was used for all assessments, and all *P* values were unadjusted.

For dose-ranging studies, only results from the dose arm closest to the label for each indication were included in the meta-analysis. In trials investigating multiple agents, data from 1 dose arm for each intervention was included in the meta-analysis and compared with the same placebo group.

Treatment-specific effects for time-to-event data were estimated as a weighted average of medians, while treatment-specific effects for proportions were computed as overall proportions with the counts. Any additional information on each endpoint provided by the original articles (eg, other comparative measures, reported *P* values vs placebo) is outlined in Supplementary Tables 3 to 11. Forest plots including all dose arms assessed are presented in Supplementary Figures 1 to 3.

## RESULTS

### Study Characteristics and Demographics

A total of 234 potential records were identified for manual review by the initial database search, and a final set of 22 articles met inclusion criteria for the meta-analysis (Supplementary Figure 4). Tables 1 and 2 provide study characteristics and participant demographics, respectively.

For episodic treatment of orolabial herpes, 4 studies corresponding to 3 articles with a total of 3412 participants were included. Participants had a median age of 37 to 43 years and were mostly women (67%–75%). In articles providing data, participants had a median 3 to 6 orolabial herpes episodes per year and a median 20 years since their first episode.

For episodic treatment of genital herpes, 8 studies with 2942 participants were included. Participants had a median age of 32 to 41 years and in 6 of 8 studies were majority female. In articles providing data, participants had a median 5 to 8 genital herpes episodes per year, with a mean or median 7 to 10 years since their first episode.

For suppressive therapy for orolabial herpes, there was only 1 article, with 98 participants. Participants had a mean age of 39 years, were mostly women (83%), and were required to have at least 4 episodes in the previous year.

For suppressive therapy for genital herpes, 10 studies with 5148 total participants were included. The median age of participants ranged from 29 to 41 years, and all studies except 1 enrolled predominantly women. Six of the 10 studies had a minimum inclusion criterion of ≥6 episodes per year prior to enrollment. In the 7 articles providing data, the number of years since the first episode ranged from 3 to 11 years.

### Episodic Treatment of Orolabial Herpes

**Figure 1.**
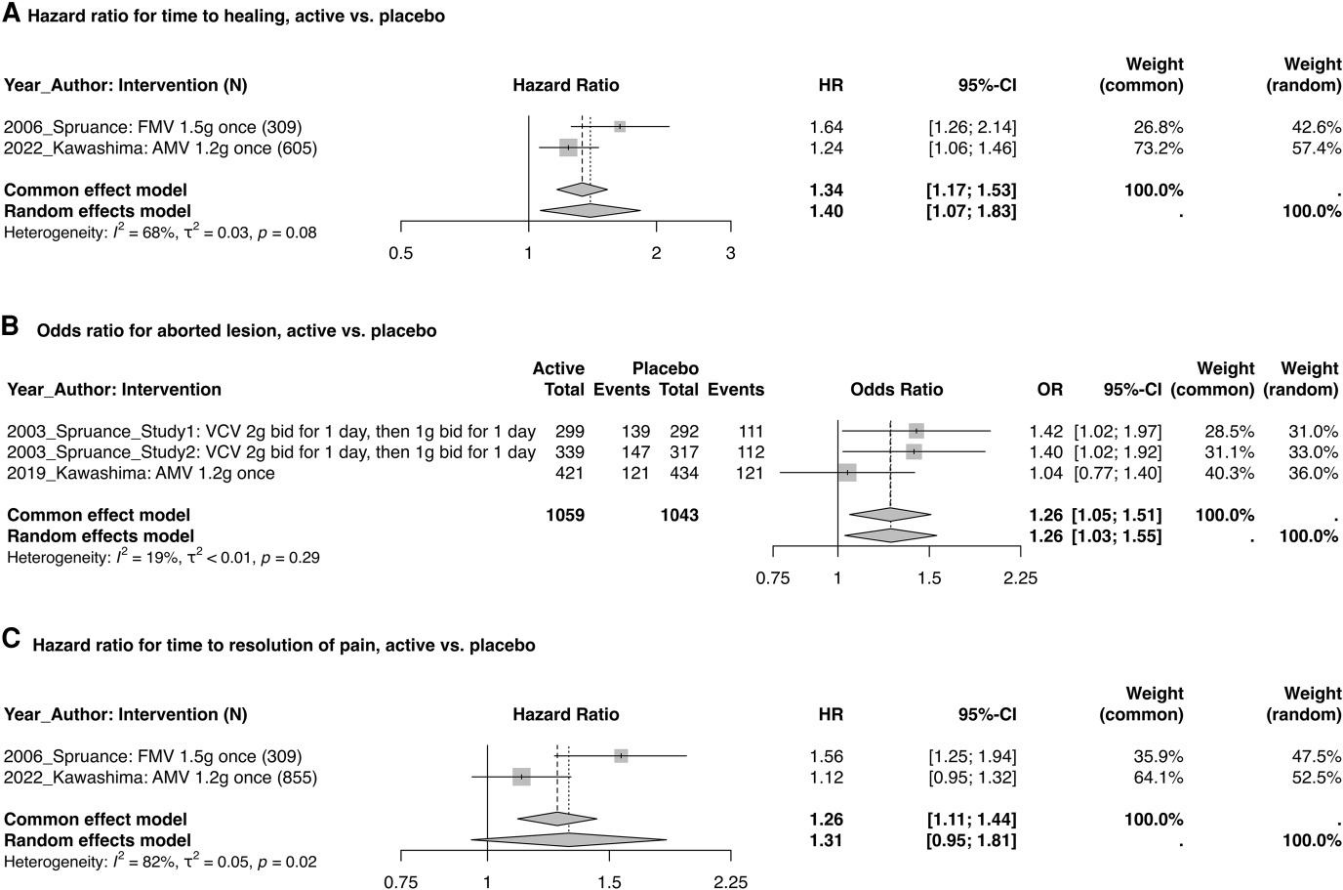
Meta-analyzed efficacy endpoints for episodic treatment of orolabial herpes. Abbreviations: AMV: amenamevir; bid, twice daily; FMV, famciclovir; HR, hazard ratio; OR, odds ratio; VCV, valacyclovir.

Four studies assessed episodic treatment of orolabial herpes with VCV, FMV, and amenamevir (Figure 1). Across the 2 studies providing data, time to healing improved from 5.7 days with placebo to 4.9 days with active intervention (difference, 0.8 days). The estimated HR (95% CI) from the random effects model for time to healing comparing active vs placebo was 1.40 (1.07–1.83), with moderate study heterogeneity (*I*^2^ = 68%, *P* = .08).

Among the 3 studies providing data, the proportion experiencing an aborted lesion improved from 33.0% with placebo to 38.4% with an active agent (difference, 5.4%). The corresponding OR (95% CI) was 1.26 (1.03–1.55) with low study heterogeneity (*I*^2^ = 19%, *P* = .29). One study assessed the proportion with an aborted lesion as an endpoint but did not report numeric results due to similarity to placebo [11].

Time to resolution of pain, assessed by 2 studies, decreased from 2.3 days with placebo to 1.9 with active intervention (difference, 0.4 days). The corresponding HR (95% CI) was 1.31 (.95–1.81) with statistically significant evidence of study heterogeneity (*I*^2^ = 82%, *P* = .02).

### Episodic Treatment of Genital Herpes

**Figure 2.**
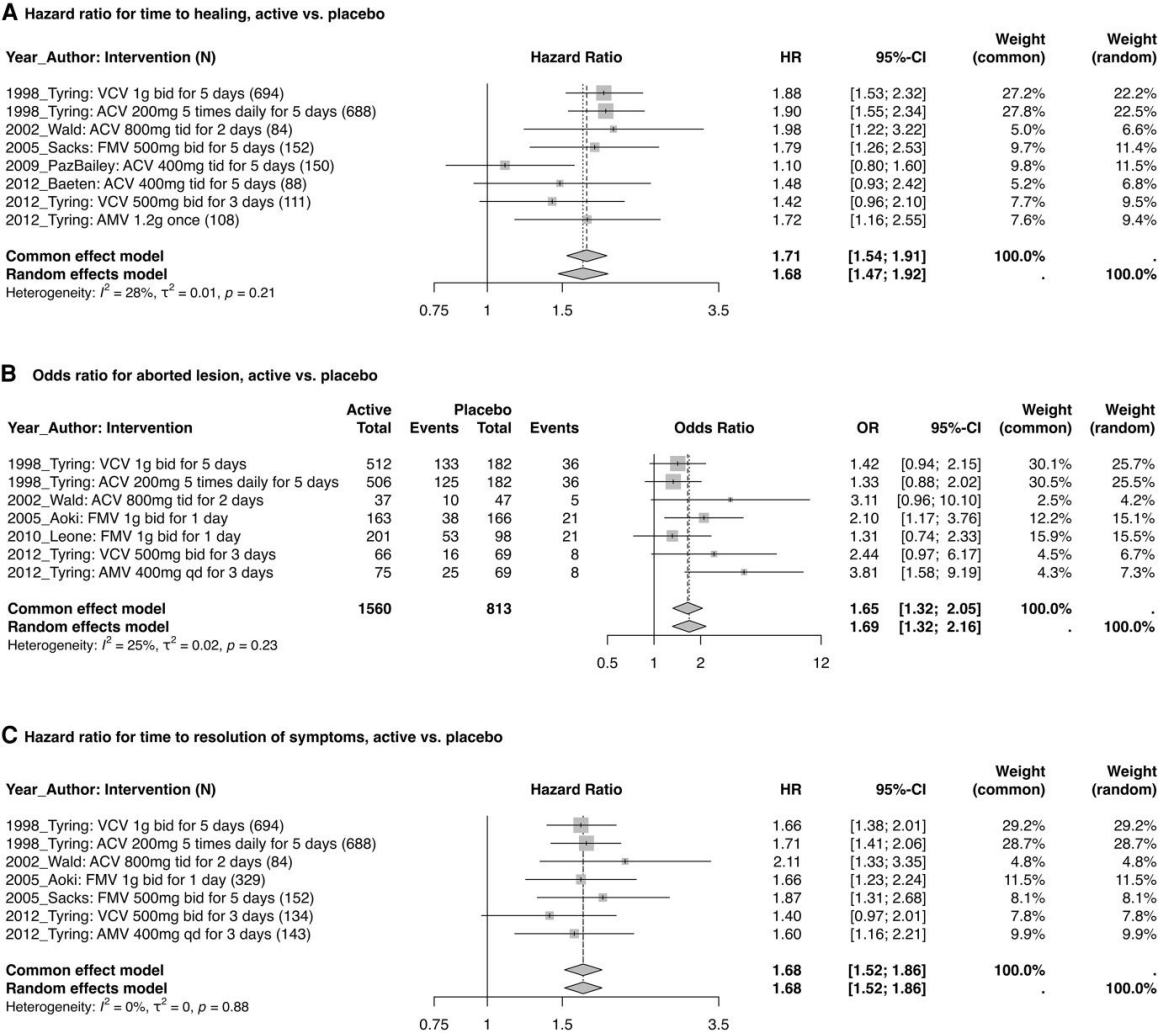
Meta-analyzed efficacy endpoints for episodic treatment of genital herpes. Abbreviations: ACV, acyclovir; AMV, amenamevir; bid, twice daily; FMV, famciclovir; HR, hazard ratio; OR, odds ratio; qd, daily; VCV, valacyclovir.

Eight studies assessed episodic treatment of genital herpes with ACV, VCV, FMV, and amenamevir (Figure 2). Across the 6 studies providing data, time to healing improved from 5.8 days with placebo to 4.7 days with active agent (difference, 1.1 days). The estimated HR (95% CI) from the random effects model for time to healing comparing active vs placebo was 1.68 (1.47–1.92), with low study heterogeneity (*I*^2^ = 28%, *P* = .21). One study reported an HR of 1.64 but no measure of variability, so it was excluded from the meta-analysis [13 ], and 1 study reported 90% CIs that were recalculated as 95% CIs [17].

Among the 5 studies providing data, the proportion experiencing an aborted lesion increased from 16.2% with placebo to 25.6% on an active agent (difference, 9.4%). The estimated OR (95% CI) was 1.69 (1.32–2.16) with some study heterogeneity (*I*^2^ = 25%, *P* = .23).

Time to resolution of symptoms, assessed by 5 studies, decreased from 5.3 days with placebo to 4.4 days on active agent (difference, 0.9 days). The estimated HR (95% CI) from the random effects model was 1.68 (1.52–1.86), with low study heterogeneity (*I*^2^ = 0%, *P* = .88).

### Suppressive Therapy for Orolabial Herpes

Only 1 article for suppressive therapy for orolabial herpes was included [20]. As a result, efficacy outcomes are briefly described here. This article presented the combined results of 2 studies comparing VCV (500 mg daily) vs placebo assessed over 16 weeks in 95 participants with a history of ≥4 episodes in the previous year. Mean time to first recurrence was longer in the VCV group as compared with placebo (13.1 vs 9.6 weeks, *P* = .016). The proportion of participants recurrence-free over the 16-week study period was higher with VCV vs placebo (60% vs 38%, *P* = .041). The number of recurrences per participant per month was 0.12 with VCV vs 0.21 with placebo (*P* = .042).

### Suppressive Therapy for Genital Herpes

**Figure 3.**
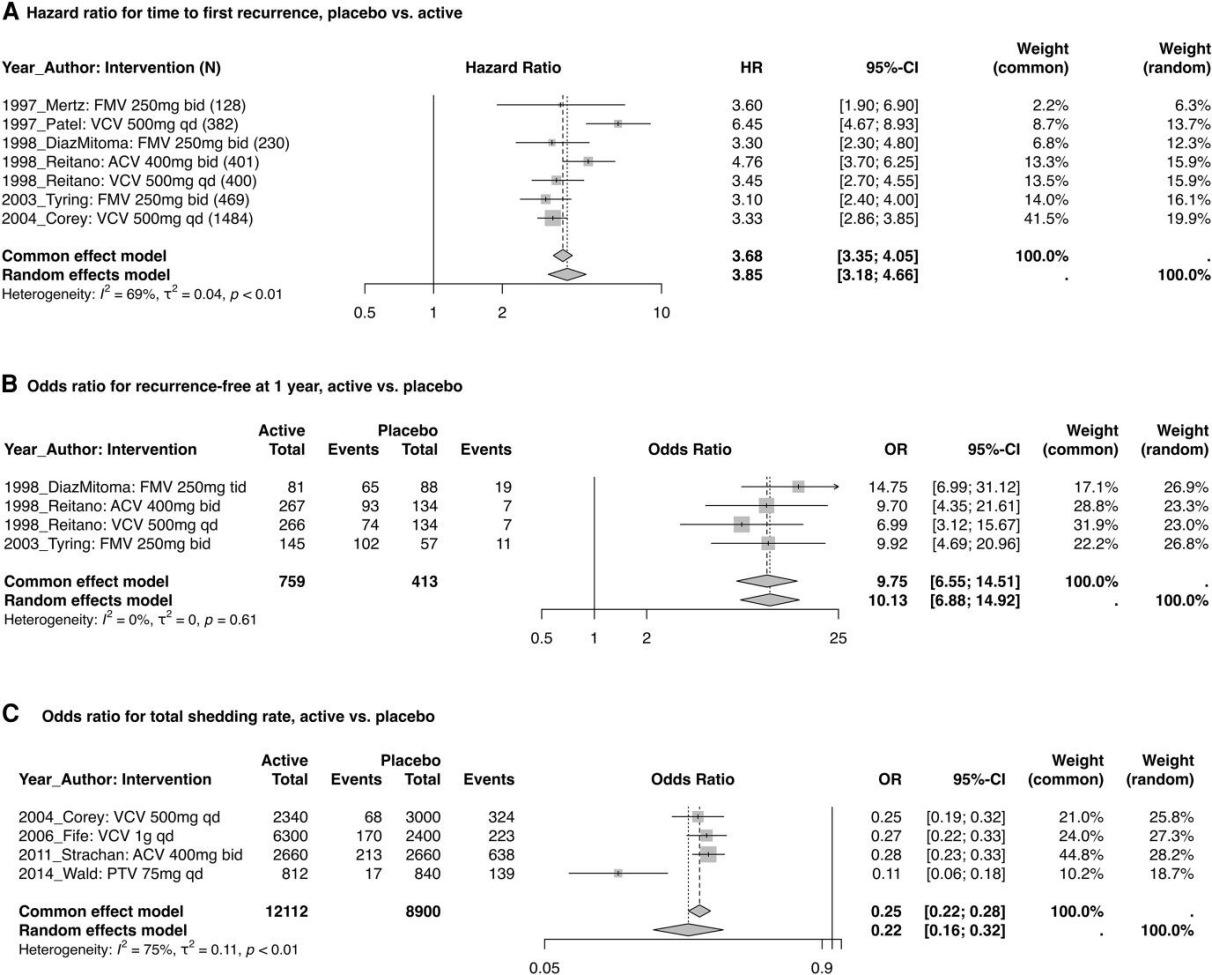
Meta-analyzed efficacy endpoints for suppressive treatment of genital herpes. Abbreviations: ACV, acyclovir; bid, twice daily; FMV, famciclovir; HR, hazard ratio; OR, odds ratio; qd, daily; VCV, valacyclovir.

Ten studies assessed suppressive therapy for genital herpes with ACV, FMV, VCV, and pritelivir (Figure 3). Across the 6 studies with data, time to first recurrence lengthened from 47 days with placebo to >225 days with active intervention (difference >178 days). The estimated HR (95% CI) from the random effects model was 3.85 (3.18–4.66), with statistically significant evidence of study heterogeneity (*I*^2^ = 69%, *P* < .01).

The proportion recurrence-free at 1 year, assessed by 3 studies, increased from 13.3% with placebo to 44.0% with an active agent (difference of 30.7%). The estimated OR (95% CI) from the random effects model was 10.13 (6.88–14.92), with no evidence of study heterogeneity (*I*^2^ = 0%, *P* = .61).

Among the 4 studies providing data, the total shedding rate was 3.9% with active vs 14.9% with placebo (difference, 11.0%). The estimated OR (95% CI) from the random effects model was 0.22 (.16–.32), with statistical evidence of study heterogeneity (*I*^2^ = 75%, *P* < .01).

## DISCUSSION

We assessed literature from 1995 to 2024 to provide a contemporary view of placebo-controlled efficacy data for orolabial and genital herpes following the licensure of antivirals such as ACV, VCV, and FMV. Few randomized placebo-controlled antiviral trials of novel agents in immunocompetent populations have been completed over the past 30 years, relatively few of which were performed in the last decade. Despite the high prevalence of orolabial and genital herpes and the implications for sexual and physical health, it has been difficult to encourage investment in new antiherpes agents.

We assessed multiple endpoints across episodic and suppressive therapies for orolabial and genital herpes to establish benchmarks for efficacy for future clinical trials. We observed different inclusion criteria across studies, heterogeneity in which endpoints were assessed and their definitions, and different quantitative assessments of the same endpoints. For example, for orolabial herpes, definitions of time to healing varied widely, with several subjective definitions ranging from the time to healing of primary lesions, the duration until lesions heal to normal-appearing skin, the time to re-epithelialization of all lesions, and the time until the loss of crust in vesicular lesions. For genital herpes, definitions included the time to healing of all nonaborted and aborted lesions, complete healing of all lesions, and cessation of HSV shedding. These differing endpoints contribute to inconsistencies in reported healing times, emphasizing the need for standardized definitions to achieve more consistent and comparable outcomes. Nevertheless, there were some similarities in the endpoints used in trials, and most individual trials showed significant benefit of treatment over placebo.

This analysis provides an opportunity to consider advantages and disadvantages of clinical trial endpoints for future trials of novel antiviral therapy for HSV (summarized in Supplementary Table 12). While the FDA has issued industry guidance for trials of recurrent herpes labialis [8], no similar guidance exists for genital herpes. The FDA-recommended outcome for orolabial herpes treatment is a half-day decrease in duration of episode, relative to control. For episodic therapy trials, time to healing and resolution of clinical symptoms are appropriate clinical endpoints, although resolution of pain is a subjective endpoint and the definition of healing may vary, as demonstrated in this analysis. In addition, antiviral agents will have greatest impact if given early in an HSV recurrence, as a period of healing will be required following tissue damage even in the presence of antiviral therapy. Thus, time to initiation of study drug may need to be incorporated into assessments. Time to virologic clearance is an objective measure of potency of the antiviral therapy, but it may not be accepted by regulators as an endpoint.

For suppressive therapy, the FDA recommends either the number of virologically confirmed recurrences or the time to first recurrence as the primary endpoint for orolabial herpes trials. While time to first virologically confirmed recurrence is an objective, easily measured outcome, it does not capture the natural history of orolabial or genital herpes, which is characterized by intermittent recurrences. Recurrence rate over a defined period is more reflective of natural history but does not account for recurrence duration, which may also be affected by antiviral therapy. Lesion rate (or days with lesions over time) is an endpoint that captures the number and duration of recurrences and has been used as an endpoint in studies of therapeutic HSV vaccines [32]. The recurrence rate and lesion rate can be virologically confirmed by self-collected swabs that can be tested for HSV polymerase chain reaction. HSV genital shedding rate is a more powerful and sensitive endpoint than clinical recurrences and has been established as a surrogate for genital herpes lesions [33]. While HSV genital shedding rate has not traditionally been accepted as a primary endpoint by regulators for licensure, it can be helpful for dose selection in early-phase studies. In addition, measures incorporating frequency and quantity of viral shedding, such as area under the curve, may provide insight into potential for virus transmission, a key concern for patients with HSV infection.

Strengths of this analysis stem from its broad scope: we assessed 4 indications for treatment of HSV across multiple endpoints and compared endpoint definitions across studies. Use of only placebo-controlled trials allowed assessment of treatment effects.

Limitations of this analysis include the inability to estimate stratified treatment effects for subgroups of interest (ie, by sex) due to limited reporting of stratified analyses in source literature. Additionally, estimates of agent-specific treatment effects were precluded by limited data and different dosing strategies across studies. We made the methodological choice to pool results from different antiviral agents under a single active-treatment category, which we believe is reasonable given the review's primary objective to evaluate endpoint performance. Reassuringly, despite heterogeneity in endpoints used, endpoint definitions, and agents, the magnitude of treatment benefit is generally consistent within each indication assessed. Another limitation of this work is the exclusion of non–English language literature, which can potentially lead to bias.

Our analysis suggests that future research should address new or standardized endpoints for clinical trials intended for episodic or chronic suppressive therapeutics for orolabial and genital herpes with input from work groups such as clinicians and patients as well as regulatory guidance. Here we have provided a first step toward establishing the need for consistent study design for evaluation of new agents in the future.

## CONCLUSION

The results of this meta-analysis demonstrate the heterogeneity of efficacy endpoints utilized in placebo-controlled trials to test antiviral agents for treatment and suppression of orolabial and genital herpes and highlight the need for further innovation, standardization, and consistency in trial design.

## Supplementary Material

ofaf776_Supplementary_Data

## Supplementary Data

Supplementary materials are available at *Open Forum Infectious Diseases* online. Consisting of data provided by the authors to benefit the reader, the posted materials are not copyedited and are the sole responsibility of the authors, so questions or comments should be addressed to the corresponding author.
