# Supplementary material for: Orolabial and Genital Herpes Clinical Trials: A Meta-analysis of Endpoints
Source: Open Forum Infect Dis. 2025 Dec 17;13(1):ofaf776. doi: 10.1093/ofid/ofaf776 (PMC12777976; doi:10.1093/ofid/ofaf776)
Supplement: ofaf776_Supplementary_Data [file ofaf776_supplementary_data.docx]

**SUPPLEMENTARY MATERIALS**

Table S1: Full search strategy* and number of identified records (Date of search: 12 March 2024)

| **Category** | **Search** | **Query** | **Number of Identified Records** |
| --- | --- | --- | --- |
| **Population** | 1 | exp *Herpes/ | 37841 |
|  | 2 | ("Herpes Zoster" or "Herpes Zoster virus" or HZV or "Herpes Simplex Virus" or HSV or "Varicella Zoster Virus" or VZV or "Herpes Labialis" or "Herpes Simplex Labialis" or shingles).ti,ab. | 336991 |
|  | **3** | **1 or 2** | **352475** |
| **Design** | **4** | **(placebo$ control$ or active control$ or placebo-control$ or random$).ti,ab** | **8936492** |
| **Intervention** | 5 | (Valacyclovir or 256U or 256U87 or "BW 256" or BW256 or "BW 256U" or Talavir or Valherpes or Valtrex or Vapridol or Virval or Zelitrex).ti,ab | 7254 |
|  | 6 | (Acyclovir or Aciclovir or "Aciclovir Lauriad" or "BA 021" or BA021 or Labiriad or Sitavig or Sitavir).ti,ab. | 58917 |
|  | 7 | (Abreva or Abrax or Herepair or Lidakol or n-Docosanol).ti,ab. | 165 |
|  | 8 | (Famciclovir or AK-120 or AK120 or BRL-42810 or BRL42810 or Famvir).ti,ab. | 4351 |
|  | 9 | (Pritelivir or AIC-316 or AIC316 or "BAY 57-1293" or BAY571293).ti,ab. | 384 |
|  | 10 | ("ABI 5366" or ABI5366 or ABI-5366).ti,ab. | 0 |
|  | **11** | **5 or 6 or 7 or 8 or 9 or 10** | **65697** |
| **Outcomes** | 12 | ("viral load" or recurrence or episodes or lesions or crusting or healing or pain or itching).mp | 14952851 |
|  | 13 | (Nephrotoxicity or hepatotoxicity or neurotoxicity or "skin toxicity").mp. | 852867 |
|  | 14 | 12 or 13 | 15708337 |
|  | **15** | **3 and 4 and 11 and 14** | **1949** |
| **Exclusions** | 16 | (p?ediatric$1 or ((pediatric* or paediatric* or children or child* or toddler* or offspring or newborn* or congenital* or infan* or baby or babies or neonat* or pre-term or preterm* or premature birth* or NICU or preschool* or pre-school* or boy or boys or girl* or school* or pubescen* or juvenile* or teen* or youth* or adolescent or adolescents or adolesc* or pre-pubesc* or prepubesc*) not adult*)).ti. | 10068221 |
|  | 17 | exp healthy volunteers/ or "healthy volunteers".mp. or "healthy patients".mp. or "healthy cohort".mp. or "healthy adults".mp. or "healthy participants".mp. or "healthy subjects".mp. or "healthy individuals".mp. [mp=ab, bo, bt, ti, hw, tn, ot, dm, mf, dv, kf, fx, dq, nm, ox, px, rx, ui, sy, ux, mx] | 3136888 |
|  | 18 | (comment$ or letter$ or editorial or note or interview or case reports).pt. or case report.mp. | 21348326 |
|  | 19 | review.pt. | 13326802 |
|  | 20 | (preclinical or pre-clinical or nonclinical or non-clinical or rat or rats or mice or mouse or murine or rabbit$ or guinea pig$ or rodent$ or dog or dogs or canine$ or puppy or puppies or mongrel$ or monkey$ or primate$ or nhp or zebrafish or rabbit or frog$ or horse$ or fish or pig or pigs or boar or porcine or chromatography or hplc or spectroscopic or spectrometry or trout or chicken or chickens or guinea-pig$ or beagle or hound or feline or cd-1 or cd1 or "balb/c" or "balb c" or sprague-dawley or wistar or long-evans or cynomolgus or squirrel$ or rhesus or minipigs or gottingen or "new zealand white" or nzw).ti,ab. | 29696520 |
|  | 21 | ("real-world" or "real world" or "real-life" or "real life" or "observation" or "clinical practise" or "observational" or Retrospective or meta-analysis or "meta analysis" or review).mp. | 30843390 |
|  | 22 | ((retrospective or observational or cohort) adj3 (study or trial)).ti,ab. | 4780828 |
|  | 23 | 16 or 17 or 18 or 19 or 20 or 21 or 22 | 87492854 |
|  | **24** | **15 not 23** | **1381** |
| **Limits** | 25 | limit 24 to english language | 1345 |
|  | 26 | limit 25 to human | 1312 |
|  | 27 | limit 26 to humans | 1301 |
|  | 28 | limit 27 to yr="1995 -Current" | 480 |
|  | **29** | **Remove duplicates from 28** | **234** |

*Search included some terms for population and intervention beyond the scope of the meta-analysis to ensure no records were systematically excluded if co-mentioned with terms of interest.

Table S2: Endpoints assessed by manuscripts included in the meta-analysis

| **Manuscript ID** | **Time to Healing** | **Proportion of Aborted Lesions** | **Time to pain free** | **Time to first recurrence** | **Proportion recurrence free** | **Time to resolution of symptoms** | **Viral Shedding** |
| --- | --- | --- | --- | --- | --- | --- | --- |
| *Episodic treatment for oro-labial herpes* | | | | | | | |
| 2003_Spruance_Study1  **Primary endpoint: median duration of episode* | Tertiary/Exploratory (Time to lesion healing) | Secondary (Proportion of subjects in whom cold sore lesion development was prevented and/or blocked) | Tertiary/Exploratory (Time to cessation of pain and/or discomfort) | Not measured | Not measured | Not measured | Not measured |
| 2003_Spruance_Study2 | Tertiary/Exploratory (Time to lesion healing) | Primary (Proportion of subjects in whom cold sore lesion development was prevented and/or blocked) | Tertiary/Exploratory (Time to cessation of pain and/or discomfort) | Not measured | Not measured | Not measured | Not measured |
| 2006_Spruance | Primary (Time to healing of primary vesicular lesions) | Secondary (Proportion of participants with aborted lesions) | Secondary (Time to resolution of pain and tenderness) | Not measured | Not measured | Not measured | Not measured |
| 2022_Kawashima | Primary (Time to healing of all herpes labialis lesions in the modified intention-to- treat population) | Secondary (Proportion of participants with aborted lesions) | Secondary (Time to resolution of pain accompanying herpes labialis) | Not measured | Not measured | Not measured | Not measured |
| *Episodic treatment for genital herpes* | | | | | | | |
| 1998_Tyring | Primary (Time to lesion healing) | Secondary (Proportion of aborted episodes) | Secondary (Duration of pain) | Not measured | Not measured | Primary (Length of episode) | Secondary (Duration of viral shedding ) |
| 2002_Wald | Primary (Duration of lesion healing time) | Secondary (Proportion of episodes with aborted lesions) | Not measured | Tertiary (Time to next recurrence) | Not measured | Primary (Duration of symptoms) | Primary (Duration of viral shedding ) |
| 2005_Aoki | Primary (Time to healing of all nonaborted genital herpes lesions) | Secondary (Proportion of participants with aborted lesions) | Not measured | Not measured | Not measured | Secondary (Time to resolution of symptoms) | Not measured |
| 2005_Sacks | Primary (Time to complete healing of original lesions) | Not measured | Not measured | Not measured | Not measured | Secondary (Time to cessation of symptoms) | Primary (Time to cessation of viral shedding) |
| 2009_PazBailey  **Co-primary: proportion healed by day 7* | Primary ((median) Time to healing) | Not measured | Not measured | Not measured | Not measured | Not measured | Secondary (Effect of acyclovir on HSV-2 shedding from ulcers) |
| 2010_Leone | Primary (Time to healing of non-aborted genital herpes lesions) | Secondary (Proportion of participants with aborted genital herpes lesion) | Not measured | Not measured | Not measured | Secondary (Time to resolution of all and specific genital herpes-associated symptoms) | Not measured |
| 2012_Baeten | Primary (Time to complete healing of all genital lesions) | Not measured | Not measured | Not measured | Not measured | Not measured | Not measured |
| 2012_Tyring | Primary (Time to lesion healing) | Secondary (Proportion of participants with aborted episodes) | Secondary (Duration of Pain) | Not measured | Not measured | Secondary (Duration of symptoms) | Secondary (Duration of viral shedding ) |
| *Suppressive therapy for oro-labial herpes* | | | | | | | |
| 2004_Baker | Not measured | Not measured | Not measured | Secondary (Mean time to first recurrence) | Primary (Proportion of participants who remained recurrence-free during the 4-month treatment period) | Not measured | Not measured |
| *Suppressive therapy for genital herpes* | | | | | | | |
| 1997_Mertz | Not measured | Not measured | Not measured | Primary (Time to first clinically or virologically confirmed recurrence) | Secondary (Proportion of participants remaining recurrence-free during the 120-day study) | Not measured | Not measured |
| 1997_Patel | Not measured | Not measured | Not measured | Primary (Time to first recurrence of genital HSV infection) | Secondary (Proportion of participants recurrence-free at 16 weeks) | Not measured | Not measured |
| 1998_DiazMitoma | Not measured | Not measured | Not measured | Primary (Time to the first recurrence of genital HSV infection) | Primary (Proportion of participants remaining free of HSV recurrence at 6 months) Secondary (Proportion of participants remaining free of HSV recurrence at 12 months) | Not measured | Not measured |
| 1998_Reitano | Not measured | Not measured | Not measured | Primary (Time to first recurrence of genital HSV infection) | Secondary (Proportion of participants remaining recurrence-free at 12 months) | Not measured | Not measured |
| 2003_Tyring | Not measured | Not measured | Not measured | Primary (Time to first clinically confirmed lesional episode) | Primary (Proportion of participants who remained free from clinical HSV recurrences (confirmed by viral culture) for at least 6 months)  Secondary (Proportion of participants who remained free from HSV recurrences at 12 months) | Not measured | Not measured |
| 2004_Corey  **Primary outcome: reduction in transmission of symptomatic genital herpes* | Not measured | Not measured | Not measured | Secondary (Time to first recurrence of genital HSV-2 in the source partner) | Not measured | Not measured | Secondary (HSV-2 mucosal shedding frequency and quantity) |
| 2004_Sacks | Not measured | Not measured | Not measured | Secondary (Time to the first asymptomatic HSV-positive culture)  Secondary (Time to first HSV-positive culture) | Not measured | Not measured | Primary (Proportion of days with asymptomatic viral shedding from any genital site)  Secondary (Proportion of days with symptomatic HSV-positive shedding) |
| 2006_Fife | Not measured | Not measured | Not measured | Secondary (Time to first genital herpes recurrence) | Not measured | Not measured | Primary (Percentage of days with total (clinical and subclinical) HSV-2 shedding) |
| 2011_Strachan  **Personality traits* | Not measured | Not measured | Not measured | Not measured | Not measured | Not measured | Secondary (Effect of acyclovir treatment on lesion frequency and viral shedding) |
| 2014_Wald | Not measured | Not measured | Not measured | Not measured | Not measured | Not measured | Primary (Rate of genital HSV shedding) |

Table S3: Reported efficacy values for each study contributing to meta-analysis of time to healing in the episodic treatment of oro-labial herpes

|  | **N** | **Mean (days)** | **Median (days)** | **Range** | **95% CI for Median** | **HR (95% CI)** | **Placebo-adjusted mean difference (95% CI)** | **Reported p-value for placebo comparison** |
| --- | --- | --- | --- | --- | --- | --- | --- | --- |
| **2003_Spruance_Study1** | | | | | | | | |
| VCV 2g bid for 1 day then 1g bid for 1 day | 299 | 5 | 4.3 | (0.7, 17.4) | NA | NA | -1.1 (-1.7, -0.5) | 0.001 |
| VCV 2g bid for 1 day | 311 | 4.8 | 4.3 | (0.2, 16.7) | NA | NA | -1.3 (-1.9, -0.7) | <0.001 |
| Placebo | 292 | 6.1 | 5.1 | (1.0, 19.1) | NA |  |  |  |
| **2003_Spruance_Study2** | | | | | | | | |
| VCV 2g bid for 1 day then 1g bid for 1 day | 339 | 5.2 | 4.6 | (1.4, 15.0) | NA | NA | -1.2 (-1.7, -0.7) | <0.001 |
| VCV 2g bid for 1 day | 298 | 5.1 | 4.8 | (1.0, 15.0) | NA | NA | -1.2 (-1.8, -0.7) | <0.001 |
| Placebo | 317 | 6.4 | 5.4 | (1.6, 20.6) | NA |  |  |  |
| **2006_Spruance** | | | | | | | | |
| FMV 1.5g once | 152 | NA | 4.4 | NA | (3.9, 5.0) | 1.64 (1.26, 2.14) | NA | <0.001 |
| FMV 750 mg bid for 1 day | 157 | NA | 4 | NA | (3.8, 4.8) | 2.05 (1.58, 2.66) | NA | <0.001 |
| Placebo | 168 | NA | 6.2 | NA | (5.7, 7.0) |  |  |  |
| **2022_Kawashima** | | | | | | | | |
| AMV 1.2g once | 298 | NA | 5.1 | NA | (4.9, 5.4) | 1.24 (1.06, 1.46) | NA | 0.0085 |
| Placebo | 307 | NA | 5.5 | NA | (5.1, 6.0) |  |  |  |

AMV: amenamevir, bid: twice daily, CI: confidence interval, FMV: famciclovir, HR: hazard ratio, NA: not applicable/not reported, VCV: valacyclovir

Table S4: Reported efficacy values for each study contributing to meta-analysis of percentage with an aborted lesion in the episodic treatment of oro-labial herpes

|  | **N** | **Fraction with aborted lesion** | **Percentage (%)** | **Difference (95% CI)** | **Reported p-value for placebo comparison** |
| --- | --- | --- | --- | --- | --- |
| **2003_Spruance_Study1** | | | | | |
| VCV 2g bid for 1 day then 1g bid for 1 day | 299 | 139/299 | 46.5 | 8.5 (0.2, 16.7) | 0.061 |
| VCV 2g bid for 1 day | 311 | 138/311 | 44.4 | 6.4 (-1.8, 14.5) | 0.096 |
| Placebo | 292 | 111/292 | 38 |  |  |
| **2003_Spruance_Study2** | | | | | |
| VCV 2g bid for 1 day then 1g bid for 1 day | 339 | 147/339 | 43.4 | 8.0 (0.3, 15.8) | 0.036 |
| VCV 2g bid for 1 day | 298 | 129/298 | 43.3 | 8.0 (-0.1, 16.0) | 0.054 |
| Placebo | 317 | 112/317 | 35.3 |  |  |
| **2022_Kawashima** | | | | | |
| AMV 1.2g once | 421 | NA | 28.7 | 0.9 (-5.2, 6.9) | 0.4193 |
| Placebo | 434 | NA | 27.9 |  |  |

AMV: amenamevir, bid: twice daily, CI: confidence interval, NA: not applicable/not reported, VCV: valacyclovir

Table S5: Reported efficacy values for each study contributing to meta-analysis of percentage with an aborted lesion in the episodic treatment of oro-labial herpes

|  | **N** | **Mean** | **Median** | **Range** | **Median difference (95% CI)** | **HR (95% CI)** | **Placebo-adjusted mean difference (95% CI)** | **Reported p-value for placebo comparison** |
| --- | --- | --- | --- | --- | --- | --- | --- | --- |
| **2003_Spruance_Study1** | | | | | | | | |
| VCV 2g bid for 1 day then 1g bid for 1 day | 299 | 2.5 | 1.3 | (0.0, 15.0) | NA | NA | -0.4 (-0.9, 0.1) | 0.008 |
| VCV 2g bid for 1 day | 311 | 2.1 | 1.2 | (0.0, 15.0) | NA | NA | -0.7 (-1.3, -0.2) | 0.009 |
| Placebo | 292 | 2.9 | 1.8 | (0.0, 15.0) |  |  |  |  |
| **2003_Spruance_Study2** | | | | | | | | |
| VCV 2g bid for 1 day then 1g bid for 1 day | 339 | 2.8 | 1.5 | (0.0, 14.8) | NA | NA | -0.3 (-0.8, 0.2) | 0.003 |
| VCV 2g bid for 1 day | 298 | 2.3 | 1.5 | (0.0, 14.8) | NA | NA | -0.8 (-1.4, -0.3) | <0.001 |
| Placebo | 317 | 3.1 | 2.2 | (0.0, 14.8) |  |  |  |  |
| **2006_Spruance** | | | | | | | | |
| FMV 1.5g once | 152 | NA | 1.7 | NA | NA | 1.56 (1.25, 1.94) | NA | <0.001 |
| FMV 750 mg bid for 1 day | 157 | NA | 2.1 | NA | NA | 1.25 (1.00, 1.56) | NA | 0.054 |
| Placebo | 168 | NA | 2.9 | NA |  |  |  |  |
| **2022_Kawashima** | | | | | | | | |
| AMV 1.2g once | 421 | NA | 2 | NA | -0.14 (-0.66, 0.38) | 1.12 (0.95, 1.32) | NA | 0.1689 |
| Placebo | 434 | NA | 2.1 | NA |  |  |  |  |

AMV: amenamevir, bid: twice daily, CI: confidence interval, FMV: famciclovir, HR: hazard ratio, NA: not applicable/not reported, VCV: valacyclovir

Table S6: Reported efficacy values for each study contributing to meta-analysis of time to healing in the episodic treatment of genital herpes

|  | **N** | **Mean (SE)** | **Median** | **IQR** | **95% CI for Median** | **HR (95% CI)** | **Reported p-value for placebo comparison** |
| --- | --- | --- | --- | --- | --- | --- | --- |
| **1998_Tyring** | | | | | | | |
| VCV 1g bid for 5 days | 512 | NA | 4.8 | NA | NA | 1.88 (1.53, 2.32) | <0.001 |
| ACV 200mg 5 times daily for 5 days | 506 | NA | 4.8 | NA | NA | 1.90 (1.55, 2.34) | <0.001 |
| Placebo | 182 | NA | 6.0 | NA | NA |  |  |
| **2002_Wald** | | | | | | | |
| ACV 800mg tid for 2 days | 37 | NA | 4 | (3, 6.5) | NA | 1.98 (1.22, 3.22) | 0.001 |
| Placebo | 47 | NA | 6 | (4.9, 9.1) | NA |  |  |
| **2005_Aoki** | | | | | | | |
| FMV 1g bid for 1 day | 163 | NA | 4.3 | NA | (3.9, 5.0) | 1.64 | <0.001 |
| Placebo | 166 | NA | 6.1 | NA | (5.0, 7.0) |  |  |
| **2005_Sacks** | | | | | | | |
| FMV 500mg bid for 5 days | 73 | NA | 3.9 | (2.9, 5.0) | NA | 1.79 (1.26, 2.53) | 0.0011 |
| FMV 250mg bid for 5 days | 78 | NA | 4 | (3.0, 5.4) | NA | 1.74 (1.23, 2.46) | 0.0017 |
| FMV 125mg bid for 5 days | 78 | NA | 4.3 | (3.0, 5.0) | NA | 1.48 (1.06, 2.08) | 0.0234 |
| Placebo | 79 | NA | 5 | (3.7, 7.5) |  |  |  |
| **2009_PazBailey** | | | | | | | |
| ACV 400mg tid for 5 days | 78 | NA | 5 | NA | (5, 6) | 1.1 (0.8, 1.6) | 0.473 |
| Placebo | 72 | NA | 6 | NA | (5, 7) |  |  |
| **2010_Leone** | | | | | | | |
| FMV 1g bid for 1 day | 201 | NA | 5.38 | NA | NA | NA | 0.416 |
| Placebo | 98 | NA | 4.79 | NA | NA | NA |  |
| **2012_Baeten** | | | | | | | |
| ACV 400mg tid for 5 days | 61 | 5.1 (0.3) | 5 | (4, 7) | NA | 1.48 (0.93, 2.42) | 0.098 |
| Placebo | 27 | 6.0 (0.6) | 5 | (4, 7) | NA |  |  |
| **2012_Tyring** | | | | | | | |
| VCV 500mg bid for 3 days | 50 | NA | 4.7 | NA | NA | 1.42 (0.96, 2.10) | 0.077 |
| AMV 1.2g once | 47 | NA | 4.3 | NA | NA | 1.72 (1.16, 2.55) | 0.007 |
| AMV 400mg qd for 3 days | 50 | NA | 4.8 | NA | NA | 1.25 (0.86, 1.84) | 0.25 |
| AMV 200mg qd for 3 days | 54 | NA | 4.4 | NA | NA | 1.40 (0.96, 2.03) | 0.081 |
| AMV 100mg qd for 3 days | 66 | NA | 5 | NA | NA | 1.40 (0.98, 2.00) | 0.065 |
| Placebo | 61 | NA | 5.8 | NA | NA |  |  |

ACV: acyclovir, AMV: amenamevir, bid: twice daily, CI: confidence interval, FMV: famciclovir, HR: hazard ratio, IQR: interquartile range, NA: not applicable/not reported, qd: daily, SE: standard error, tid: three times daily, VCV: valacyclovir

Table S7: Reported efficacy values for each study contributing to meta-analysis of percentage with an aborted lesion in the episodic treatment of genital herpes

|  | **N** | **Fraction with aborted lesion** | **Percentage (%)** | **Proportion Difference (95% CI)** | **Reported p-value for placebo comparison** |
| --- | --- | --- | --- | --- | --- |
| **1998_Tyring** | | | | | |
| VCV 1g bid for 5 days | 512 | NA | 25.9 | 6.1 (-0.7, 13.1) | NA |
| ACV 200mg 5 times daily for 5 days | 506 | NA | 25.8 | 5.0 (-2.0, 11.8) | NA |
| Placebo | 182 | NA | 19.8 |  |  |
| **2002_Wald** | | | | | |
| ACV 800mg tid for 2 days | 37 | 10/37 | 27 | 16.4 (1.0, 31.8) | 0.029 |
| Placebo | 47 | 5/47 | 10.6 |  |  |
| **2005_Aoki** | | | | | |
| FMV 1g bid for 1 day | 163 | 38/163 | 23.3 | 10.6 (2.4, 18.8) | 0.003 |
| Placebo | 166 | 21/166 | 12.7 |  |  |
| **2010_Leone** | | | | | |
| FMV 1g bid for 1 day | 201 | NA | 26.2 | 4.5 (-7.4, 16.4) | 0.147 |
| Placebo | 98 | NA | 21.7 |  |  |
| **2012_Tyring** | | | | | |
| VCV 500mg bid for 3 days | 66 | 16/66 | 24.2 | 12.6 (0.0, 25.2) | 0.03 |
| AMV 1.2g qd for 1 day | 63 | 16/63 | 25.4 | 13.8 (1.0, 26.6) | 0.05 |
| AMV 400mg qd for 3 days | 75 | 25/75 | 33.3 | 21.7 (8.3, 35.1) | 0.002 |
| AMV 200mg qd for 3 days | 73 | 19/73 | 26 | 14.4 (1.6, 27.2) | 0.04 |
| AMV 100mg qd for 3 days | 79 | 13/79 | 16.5 | 4.9 (-0.07, 16.5) | 0.45 |
| Placebo | 69 | 8/69 | 11.6 |  |  |

ACV: acyclovir, AMV: amenamevir, bid: twice daily, CI: confidence interval, FMV: famciclovir, qd: daily, tid: three times daily, VCV: valacyclovir

Table S8: Reported efficacy values for each study contributing to meta-analysis of time to resolution of symptoms in the episodic treatment of genital herpes

|  | **N** | **Mean** | **Median** | **95% CI for Median** | **HR (95% CI)** | **Reported p-value for placebo comparison** |
| --- | --- | --- | --- | --- | --- | --- |
| **1998_Tyring** | | | | | | |
| VCV 1g bid for 5 days | 512 | NA | 4.8 | NA | 1.88 (1.53, 2.32) | <0.001 |
| ACV 200mg 5 times daily for 5 days | 506 | NA | 4.8 | NA | 1.90 (1.55, 2.34) | <0.001 |
| Placebo | 182 | NA | 5.9 | NA |  |  |
| **2002_Wald** | | | | | | |
| ACV 800mg tid for 2 days | 37 | NA | 4 | NA | 2.11 (1.33, 3.35) | <0.001 |
| Placebo | 47 | NA | 6 | NA |  |  |
| **2005_Aoki** | | | | | | |
| FMV 1g bid for 1 day | 163 | NA | 3.3 | (2.8, 4.1) | 1.66 (1.23, 2.24) | <0.001 |
| Placebo | 166 | NA | 5.4 | (4.5, 6.5) |  |  |
| **2005_Sacks** | | | | | | |
| FMV 500mg bid for 5 days | 73 | NA | 2.9 | NA | 1.87 (1.31, 2.68) | 0.0006 |
| FMV 250mg bid for 5 days | 78 | NA | 3 | NA | 1.91 (1.34, 2.73) | 0.0004 |
| FMV 125mg bid for 5 days | 78 | NA | 3.3 | NA | 1.86 (1.30, 2.67) | 0.0008 |
| Placebo | 79 | NA | 3.8 | NA |  |  |
| **2010_Leone** | | | | | | |
| FMV 1g bid for 1 day | 201 | NA | 4.5 | NA | NA | 0.329 |
| Placebo | 98 | NA | 5.7 | NA |  |  |
| **2012_Tyring** | | | | | | |
| VCV 500mg bid for 3 days | 66 | NA | 3 | NA | 1.4 (0.97, 2.01) | 0.06 |
| AMV 1.2g qd for 1 day | 63 | NA | 4.2 | NA | 1.1 (0.83, 1.47) | 0.76 |
| AMV 400mg qd for 3 days | 75 | NA | 3.3 | NA | 1.6 (1.16, 2.21) | 0.01 |
| AMV 200mg qd for 3 days | 72 | NA | 2.8 | NA | 1.3 (0.94, 1.79) | 0.14 |
| AMV 100mg qd for 3 days | 80 | NA | 2.7 | NA | 1.5 (1.06, 2.11) | 0.01 |
| Placebo | 68 | NA | 4.5 | NA |  |  |

ACV: acyclovir, AMV: amenamevir, bid: twice daily, CI: confidence interval, FMV: famciclovir, HR: hazard ratio, NA: not applicable/not reported, VCV: valacyclovir

Table S9: Reported efficacy values for each study contributing to meta-analysis of time to first recurrence in the suppressive treatment of genital herpes

|  | **N** | **Median (days)** | **HR (95% CI)** | **Reported p-value for placebo comparison** |
| --- | --- | --- | --- | --- |
| **1997_Mertz** | | | | |
| FMV 500mg qd | 61 | >120 | 1.7 (1.0, 2.8) | 0.06 |
| FMV 250mg bid | 64 | >120 | 3.6 (1.9, 6.9) | <0.001 |
| FMV 250mg qd | 61 | >120 | 1.6 (1.0, 2.8) | 0.07 |
| FMV 125mg bid | 65 | >120 | 1.8 (1.0, 3.0) | 0.03 |
| FMV 125mg qd | 60 | 114 | 1.4 (0.8, 2.3) | 0.22 |
| Placebo | 64 | 82 |  |  |
| **1997_Patel** | | | | |
| VCV 500mg qd | 288 | >112 | 6.45 (4.67, 8.93) | <0.0001 |
| Placebo | 94 | 20 |  |  |
| **1998_DiazMitoma** | | | | |
| FMV 250mg tid | 113 | 307 | 3.2 (2.2, 4.6) | <0.001 |
| FMV 250mg bid | 116 | 336 | 3.3 (2.3, 4.8) | <0.001 |
| FMV 125mg tid | 112 | 222 | 2.9 (2.0, 4.0) | <0.001 |
| Placebo | 114 | 47 |  |  |
| **1998_Reitano** | | | | |
| ACV 400mg bid | 267 | 406 | 4.76 (3.70, 6.25) | NA |
| VCV 250mg bid | 274 | 403 | 4.76 (3.70, 6.25) | <0.0001 |
| VCV 1g qd | 269 | 370 | 4.55 (3.45, 5.88) | <0.0001 |
| VCV 500mg qd | 266 | 201 | 3.45 (2.70, 4.55) | <0.0001 |
| VCV 250mg qd | 269 | 118 | 2.17 (1.69, 2.78) | <0.0001 |
| Placebo | 134 | 27 |  |  |
| **2003_Tyring** | | | | |
| FMV 250mg bid | 236 | >365 | 3.1 (2.4, 4.0) | <0.0001 |
| Placebo | 233 | 59 |  |  |
| **2004_Corey** | | | | |
| VCV 500mg qd | 743 | NA | 3.33 (2.86, 3.85) | <0.001 |
| Placebo | 741 | NA |  |  |
| **2006_Fife** | | | | |
| VCV 1g qd | 109 | >60 | NA | <0.001 |
| Placebo | 43 | 46 |  |  |

ACV: acyclovir, bid: twice daily, CI: confidence interval, FMV: famciclovir, HR: hazard ratio, NA: not applicable/not reported, VCV: valacyclovir

Table S10: Reported efficacy values for each study contributing to meta-analysis of proportion recurrence-free at 1 year in the suppressive treatment of genital herpes

|  | **N** | **Count** | **Percent** | **Percent difference (95% CI)** | **Reported p-value for placebo comparison** |
| --- | --- | --- | --- | --- | --- |
| **1998_DiazMitoma** | | | | | |
| FMV 250mg tid | 81 | 65 | 80.2 | 58.6 (45.7, 70.3) | NA |
| FMV 250mg bid | 90 | 65 | 72.2 | 50.6 (37.3, 62.7) | NA |
| FMV 125mg tid | 77 | 55 | 71.4 | 49.8 (35.7, 62.3) | NA |
| Placebo | 88 | 19 | 21.6 |  |  |
| **1998_Reitano** | | | | | |
| ACV 400mg bid | 267 | NA | 35 | 30 (23, 37) | <0.001 |
| VCV 250 mg bid | 274 | NA | 34 | 29 (22, 36) | <0.001 |
| VCV 1g qd | 269 | NA | 34 | 29 (22, 36) | <0.001 |
| VCV 500 mg qd | 266 | NA | 28 | 23 (17, 30) | <0.001 |
| VCV 250mg qd | 269 | NA | 15 | 10 (4, 16) | 0.03 |
| Placebo | 134 | NA | 5 |  |  |
| **2003_Tyring** | | | | | |
| FAM 250mg bid | 145 | NA | 70 | 50 (37, 63) | <0.001 |
| Placebo | 57 | NA | 20 |  |  |

bid: twice daily, CI: confidence interval, FMV: famciclovir, NA: not applicable/not reported, VCV: valacyclovir

Table S11: Reported efficacy values for each study contributing to meta-analysis of shedding rate in the suppressive treatment of genital herpes

|  | **Days of swabbing** | **N participants** | **Proportion of daily swabs positive** | **Difference in proportion vs. placebo** | **Reported p-value for placebo comparison** |
| --- | --- | --- | --- | --- | --- |
| **2004_Corey** | | | | | |
| VCV 500mg qd | 60 | 39 | 0.029 | 0.079 | <0.001 |
| Placebo |  | 50 | 0.108 |  |  |
| **2006_Fife** | | | | | |
| VCV 1g qd | 60 | 105 | 0.027 | 0.066 | <0.001 |
| Placebo |  | 40 | 0.093 |  |  |
| **2011_Strachan** | | | | | |
| ACV 400mg bid | 140 | 19 | 0.08 | 0.16 | <0.001 |
| Placebo |  | 19 | 0.24 |  |  |
| **2014_Wald** | | | | | |
| PTV 400mg weekly | 28 | 31 | 0.053 | 0.113 | <0.001 |
| PTV 75mg qd |  | 29 | 0.021 | 0.145 | <0.001 |
| PTV 25mg qd |  | 32 | 0.093 | 0.073 | 0.06 |
| PTV 5mg qd |  | 33 | 0.182 | -0.016 | 0.7 |
| Placebo |  | 30 | 0.166 |  |  |

ACV: acyclovir, bid: twice daily, PTV: pritelivir, qd: daily, VCV: valacyclovir

Table S12: Advantages and disadvantages of endpoints used in clinical trials of episodic and suppressive therapies

| **Episodic Therapy** | | |
| --- | --- | --- |
| **Endpoint** | **Advantages** | **Disadvantages** |
| Time to healing or time to resolution of pain or symptoms | - Clinically meaningful | - Definition may be subjective, variability in measurement - Requires prompt treatment to maximize impact of antiviral intervention |
| Aborted lesion | - Clinically meaningful | - Definition may be subjective - variability in measurement |
| Time to virologic clearance | - Easily measured - Objective | - May not be accepted by regulators as primary clinical endpoint |
| **Suppressive Therapy** | | |
| **Endpoint** | **Advantages** | **Disadvantages** |
| Time to first recurrence and recurrence free at 1 year | - Objective measure - Easily measured | - Does not capture natural history of recurrences |
| Shedding rate | - Objective measure - Surrogate for clinical outcome (lesions) - Sensitive measure, allows for smaller sample size | - May not be accepted by regulators as primary clinical endpoint |
| Lesion rate (days with lesions)* | - Captures natural history of recurrences - Clinically meaningful - Virologic confirmation available | - None noted |
| Recurrence rate  (number of recurrences) | - Captures natural history of recurrences - Clinically meaningful - Virologic confirmation available | - None noted |

*Endpoint utilized in studies of therapeutic HSV vaccines

Figure S1: Forest plots for efficacy endpoints for episodic treatment of oro-labial herpes

| 1. Hazard ratio for time to healing, active vs. placebo   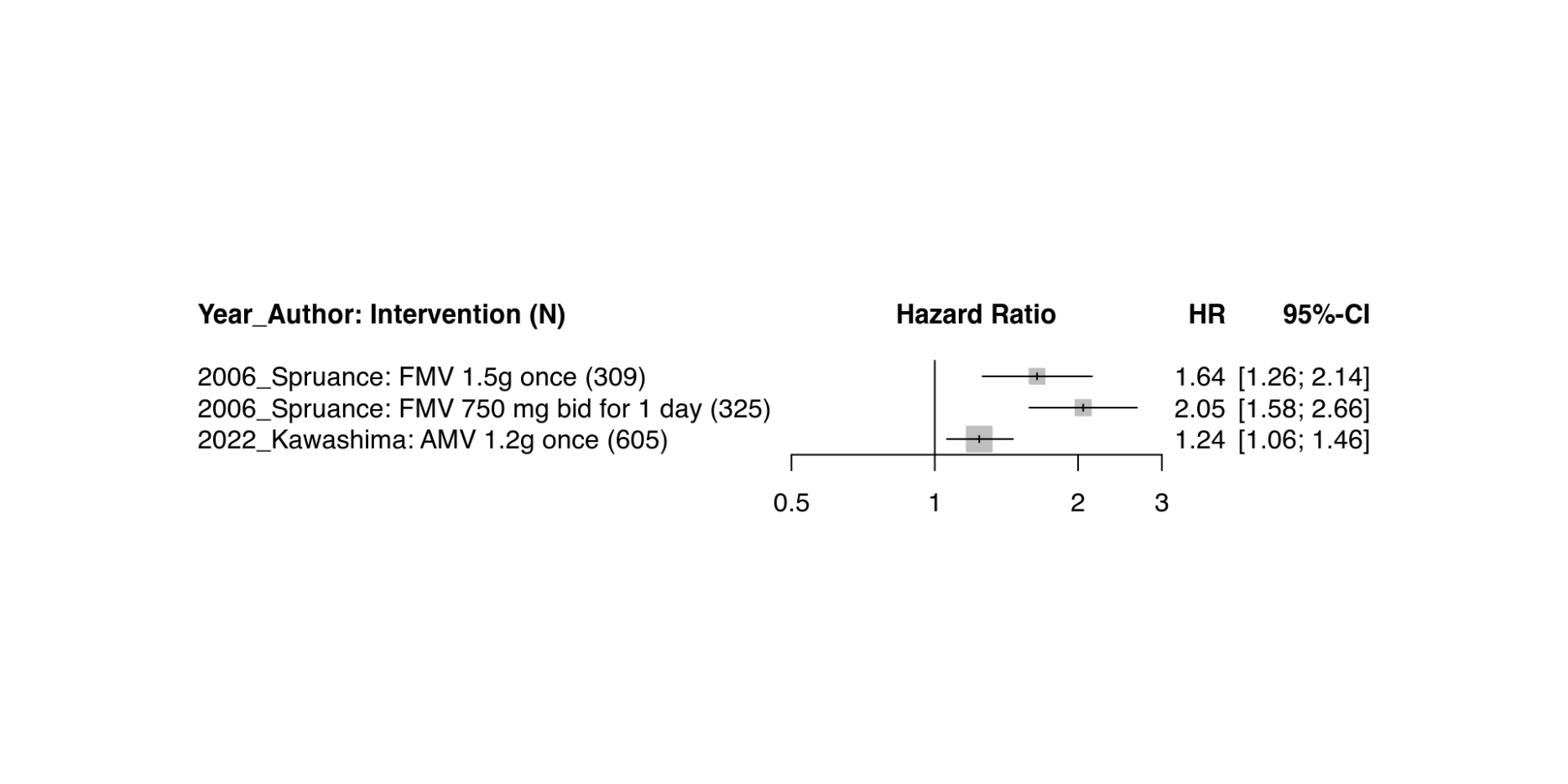 |
| --- |
| 1. Odds ratio for aborted lesion, active vs. placebo   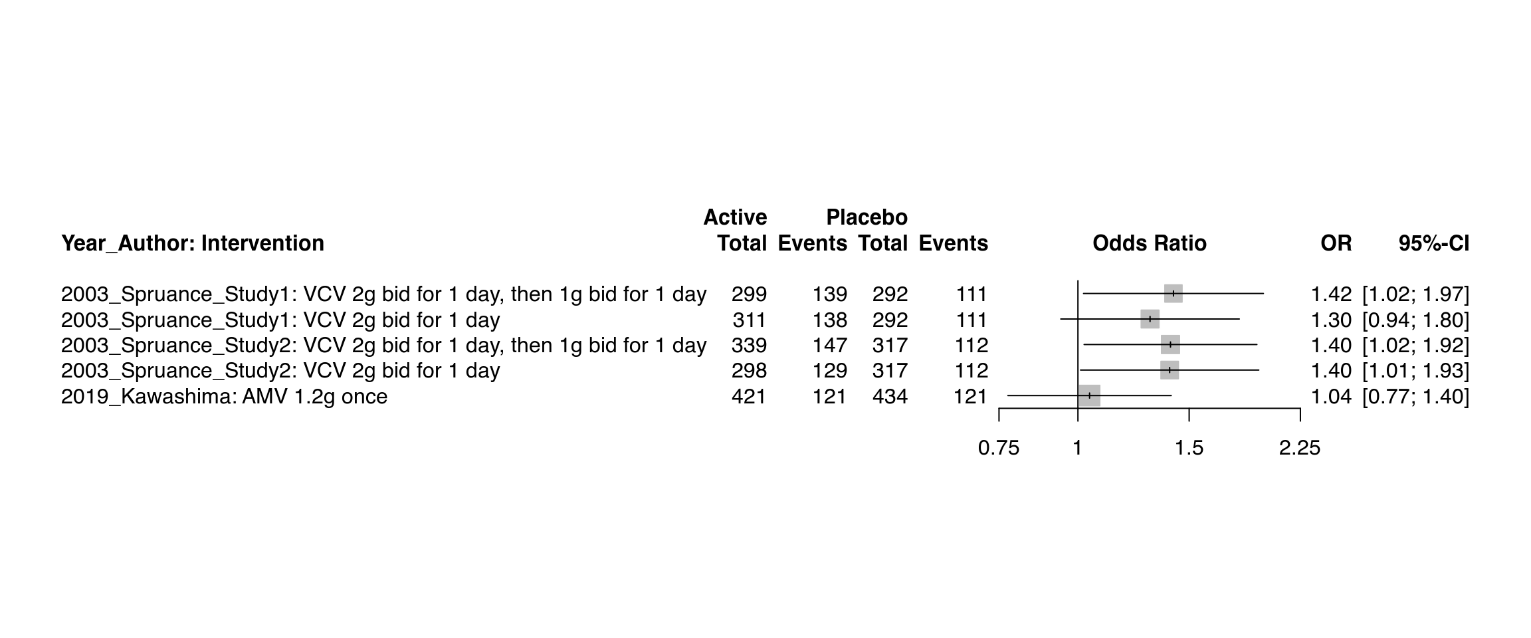 |
| 1. Hazard ratio for time to resolution of pain, active vs. placebo   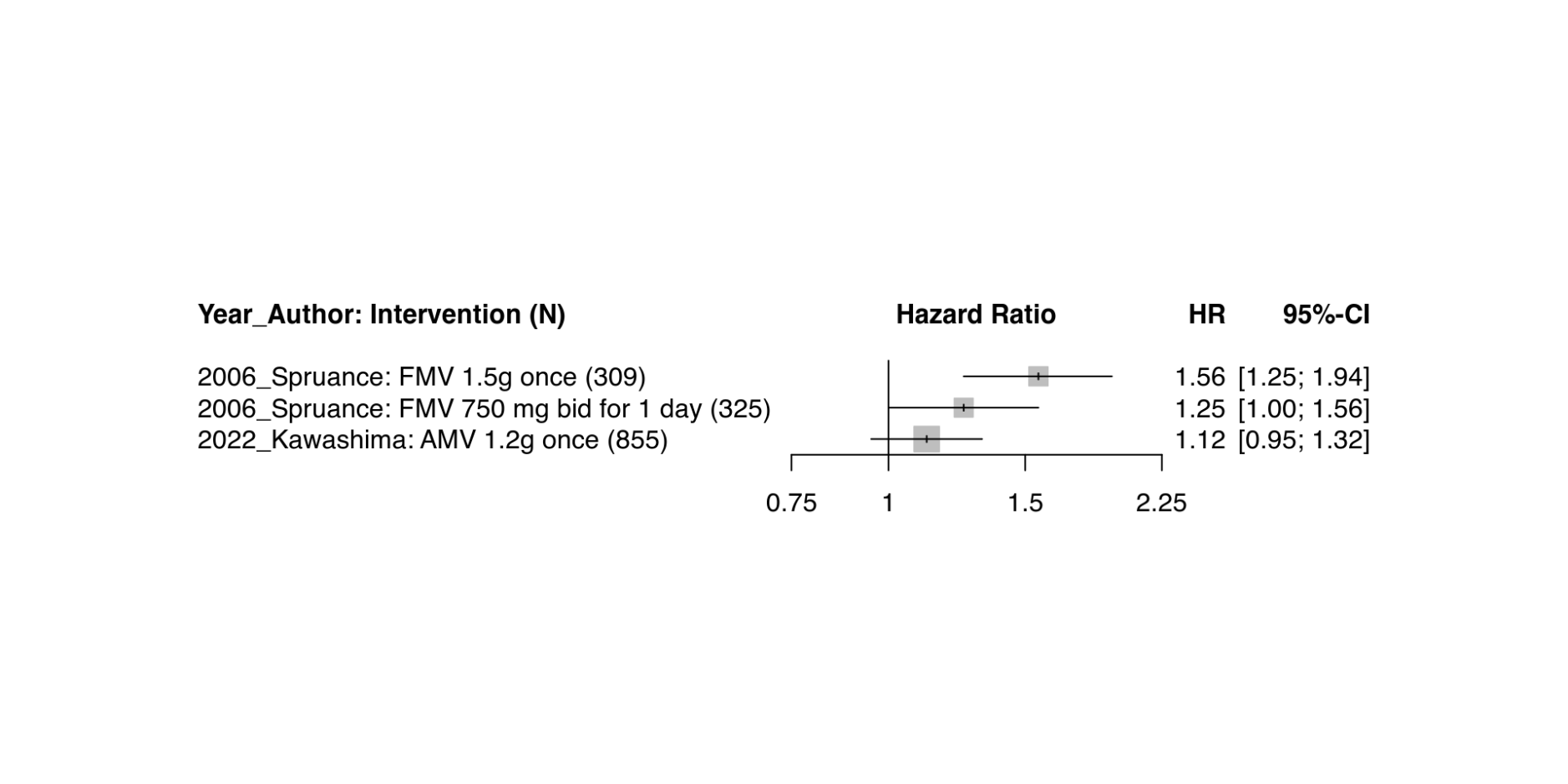 |

AMV: amenamevir, bid: twice daily, CI: confidence interval, FMV: famciclovir, HR: hazard ratio, OR: odds ratio, VCV: valacyclovir

Figure S2: Forest plots for endpoints for episodic treatment of genital herpes

| (a) Hazard ratio for time to healing, active vs. placebo  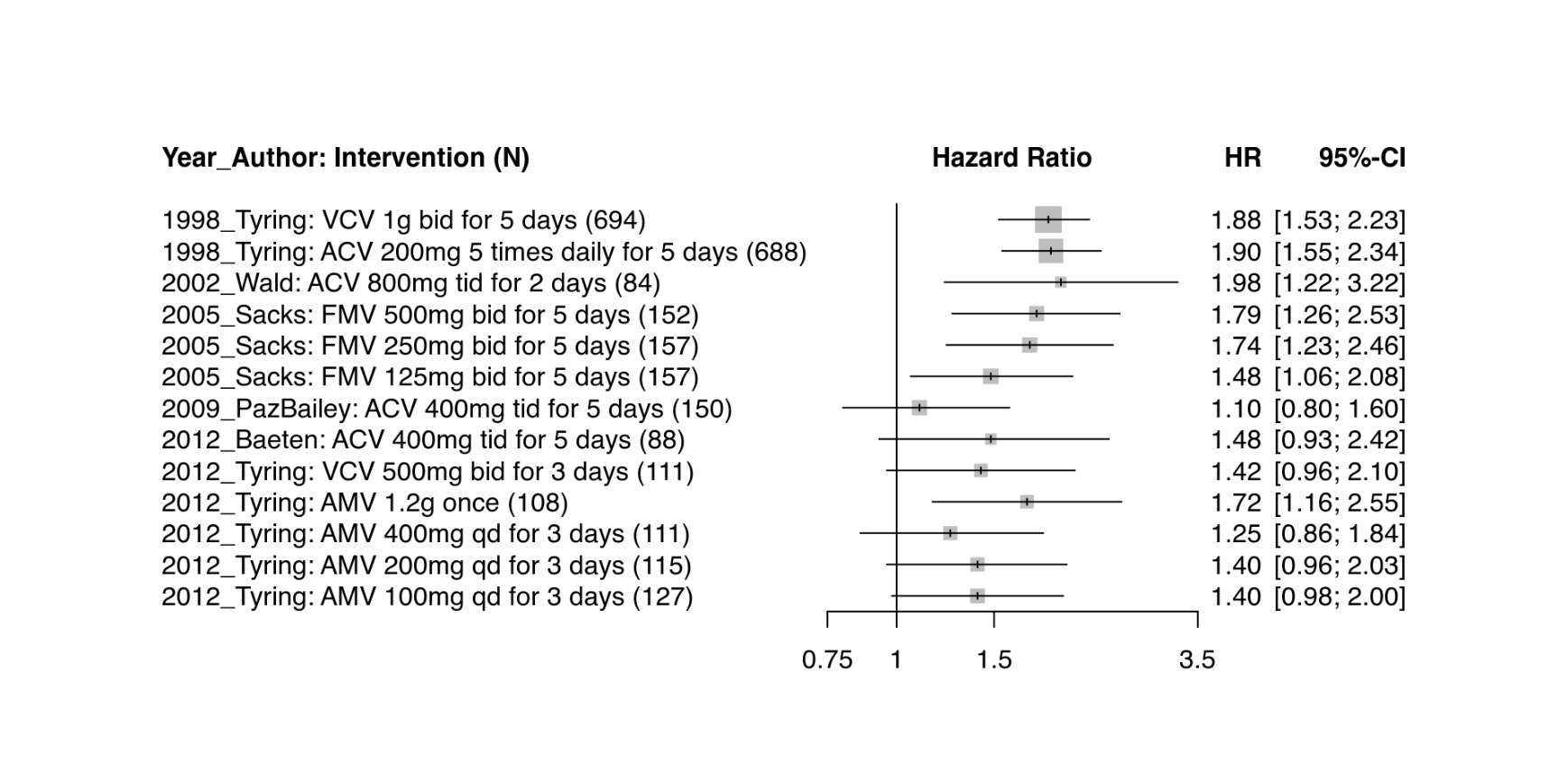 |
| --- |
| (b) Odds ratio for aborted lesions, active vs. placebo  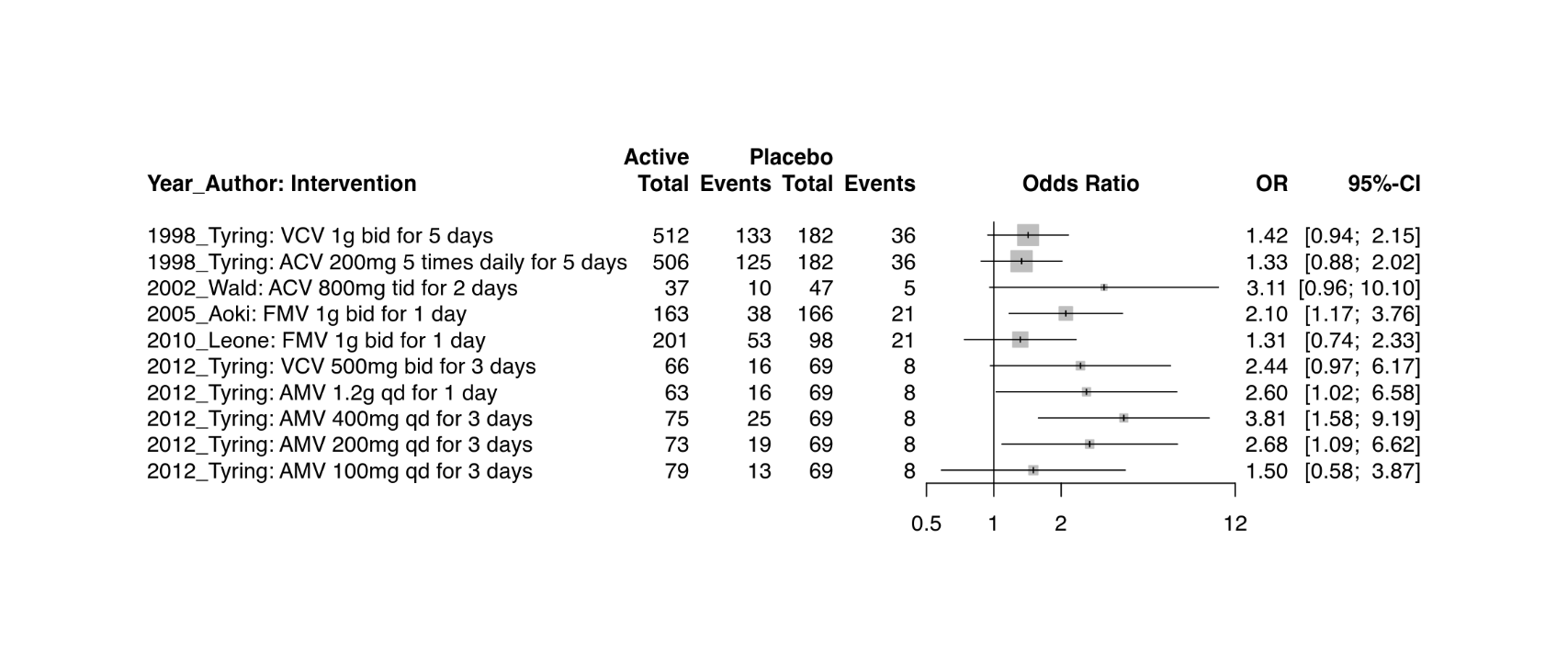 |
| (c) Hazard ratio for time to resolution of symptoms  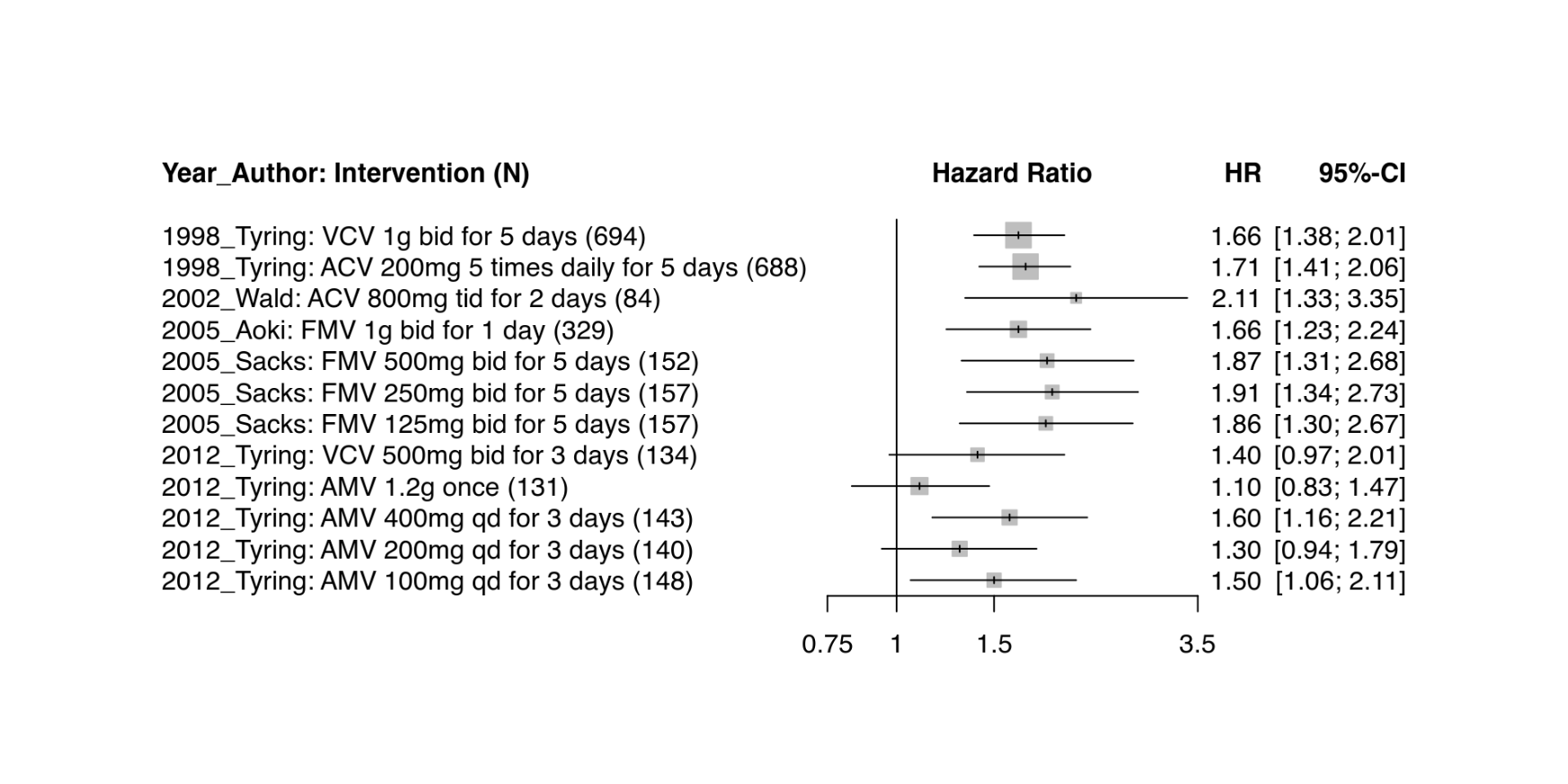 |

ACV: acyclovir, AMV: amenamevir, bid: twice daily, CI: confidence interval, FMV: famciclovir, HR: hazard ratio, OR: odds ratio, qd: daily, tid: three times daily, VCV: valacyclovir

Figure S3: Forest plots for efficacy endpoints for suppressive treatment of genital herpes

| (a) Hazard ratio for time to first recurrence, placebo vs. active  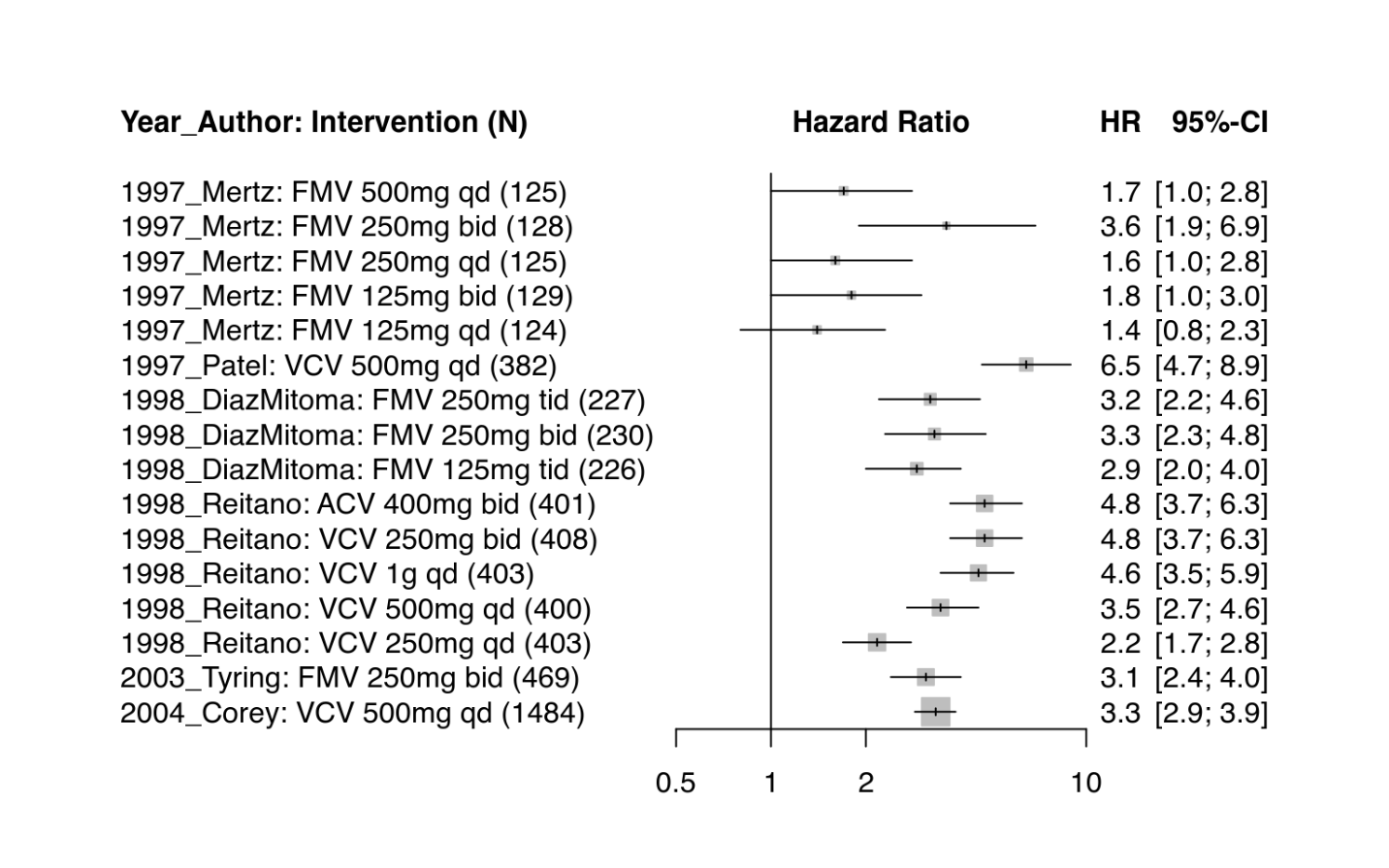 |
| --- |
| (b) Odds ratio for recurrence-free at 1 year, active vs. placebo  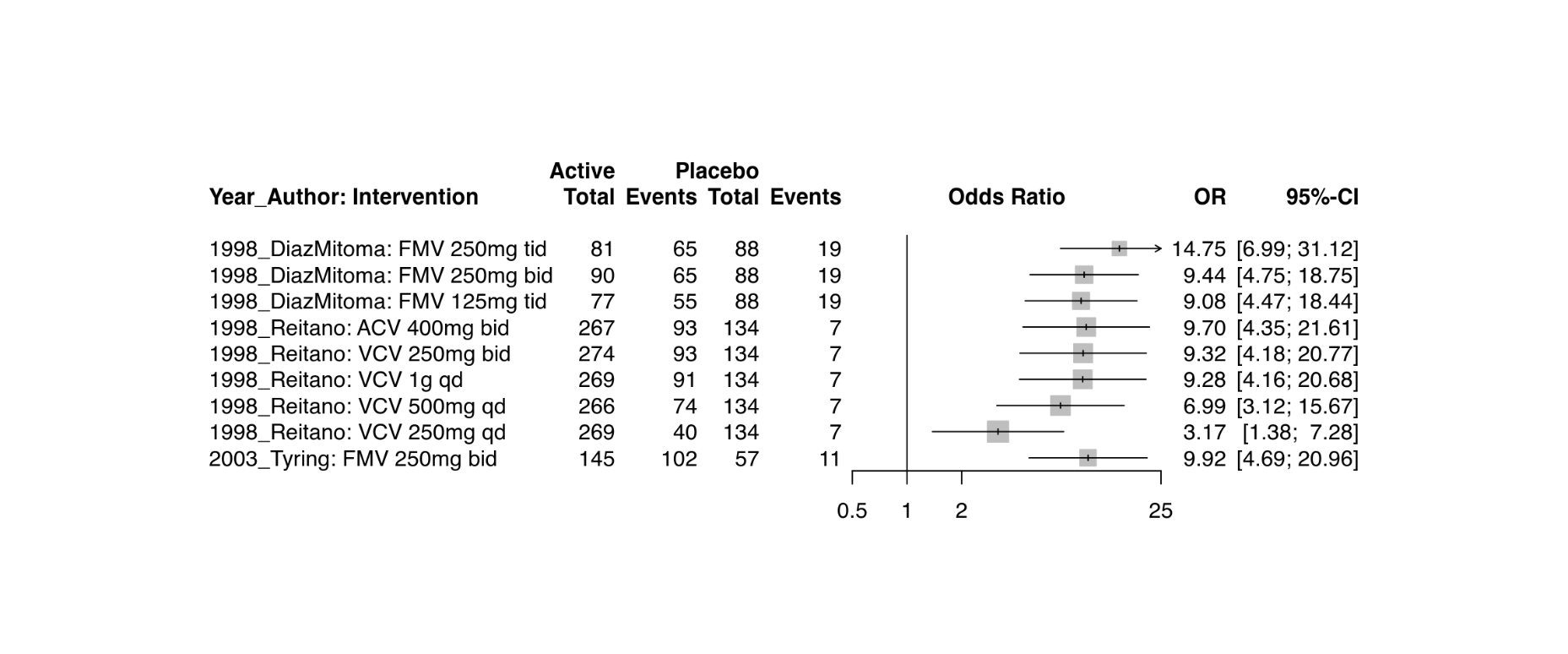 |
| (c ) Odds ratio for total shedding rate, active vs. placebo  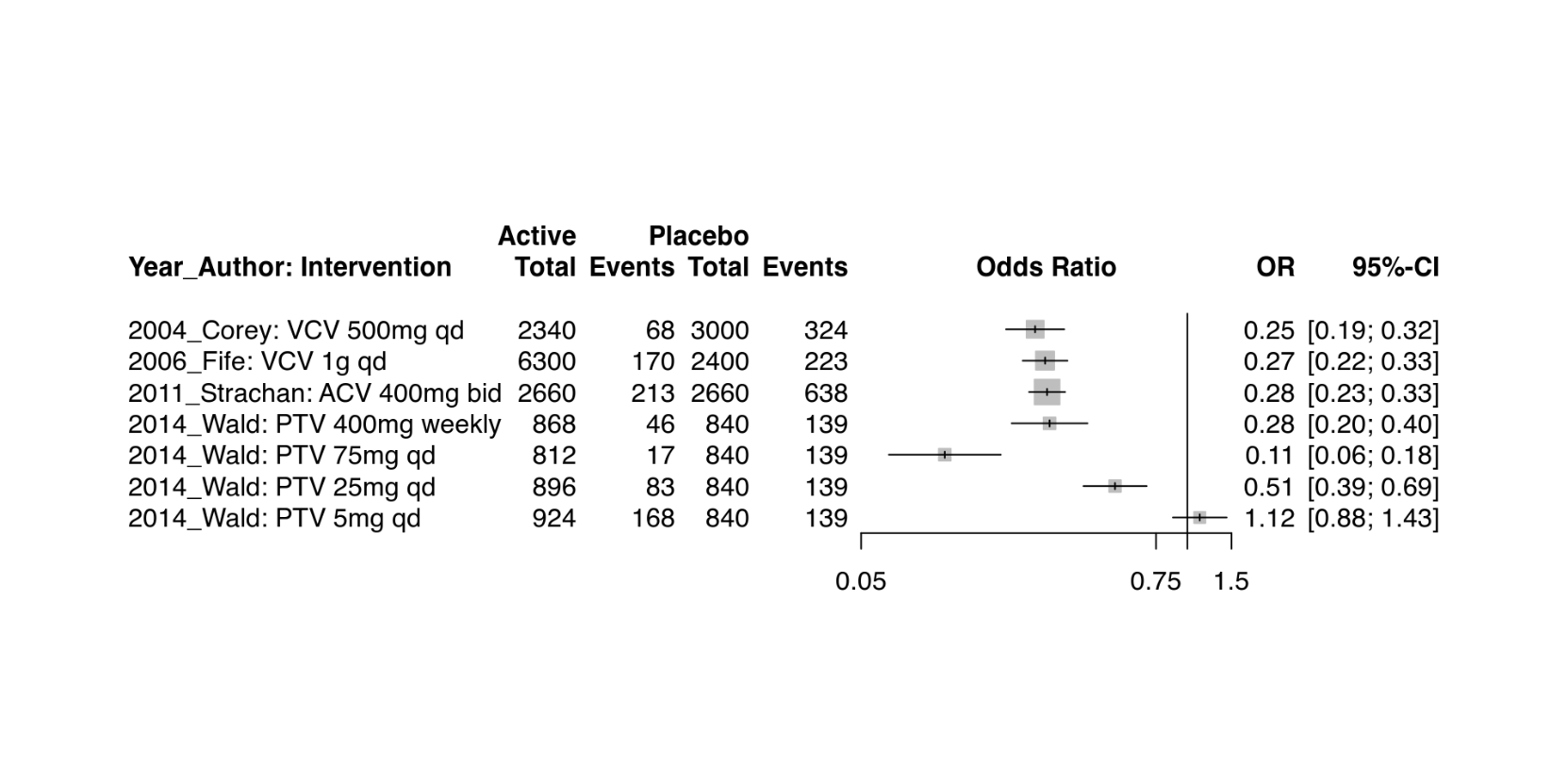 |

ACV: acyclovir, bid: twice daily, CI: confidence interval, FMV: famciclovir, HR: hazard ratio, OR: odds ratio, qd: daily, tid: three times daily, VCV: valacyclovir

Figure S4: Flow Chart of study identification, inclusion, and exclusion


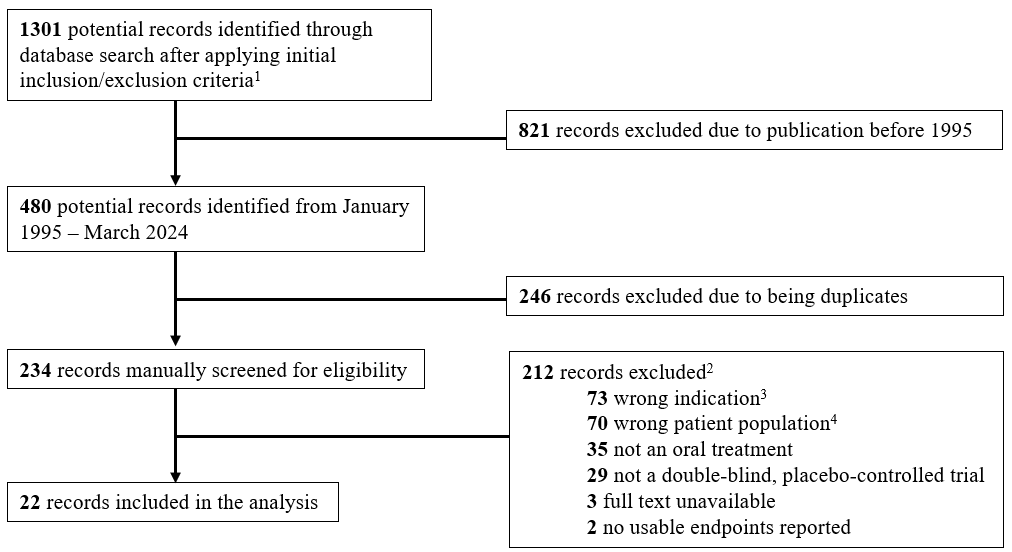


^1^See Supplementary Table S1 for full search strategy.

^2^Excluded records are only counted once but some records met multiple exclusion criteria.

^3^‘Wrong indication’ includes studies of the wrong herpes virus or wrong site (e.g, herpes zoster or herpetic eye disease).

^4^‘Wrong patient population’ includes patients with HIV and HCV and who are immunocompromised, pregnant, newly diagnosed with HSV, or not otherwise considered healthy.

HCV: hepatitis C virus, HIV: human immunodeficiency virus, HSV: herpes simplex virus
